# Pt_1_ enhanced C-H activation synergistic with Pt_n_ catalysis for glycerol cascade oxidation to glyceric acid

**DOI:** 10.1038/s41467-022-33038-w

**Published:** 2022-09-17

**Authors:** Zhe An, Zilong Zhang, Zeyu Huang, Hongbo Han, Binbin Song, Jian Zhang, Qi Ping, Yanru Zhu, Hongyan Song, Bin Wang, Lirong Zheng, Jing He

**Affiliations:** 1 State Key Laboratory of Chemical Resource Engineering, Beijing University of Chemical Technology, Beijing, 100029 P. R. China; 2 Beijing Research Institute of Chemical Industry, Sinopec Group, 100013 Beijing, P. R. China; 3 Technology Institute of High Energy Physics, Chinese Academy of Sciences, Beijing, 100049 P. R. China

**Keywords:** Heterogeneous catalysis, Catalytic mechanisms, Chemical engineering

## Abstract

The selective oxidation of glycerol to glyceric acid, an important value-added reaction from polyols, is a typical cascade catalytic process. It is still of great challenge to simultaneously achieve high glycerol activity and glyceric acid selectivity, suffering from either deep oxidation and C-C cleavage or poor oxidation efficiency from glyceraldehyde to glyceric acid. Herein, this work, inspired by nature, proposes a cascade synergistic catalysis strategy by atomic and low-coordinated cluster Pt on well-defined Cu-CuZrO_x_, which involves enhanced C-H activation on atomic Pt_1_ and O-H activation on cluster Pt_n_ in the oxidation of glycerol to glyceraldehyde, and cluster Pt_n_ for C=O activation followed by O-H insertion and atomic Pt_1_ for C-H activation in the tandem oxidation of glyceraldehyde to glyceric acid. The enhanced C-H activation in the cascade process by atomic Pt_1_ is revealed to be essential for the high glycerol activity (90.0±0.1%) and the glyceric acid selectivity (80.2±0.2%).

## Abstract (web summary)

The selective oxidation of glycerol to glyceric acid is an important value-added cascade catalytic process. Here the authors report a cascade synergistic catalysis strategy by atomic and low-coordinated cluster Pt on well-defined Cu-CuZrOx to simultaneously achieve high activity and selectivity.

## Introduction

Effective construction of chemical bonds and/or efficient synthesis of functional compounds usually requires multiple elementary steps or even cascade reactions^1–3^. Natural biological systems effectively produce elaborate molecules in the form of cascade reactions by multiple biocatalysts^4,5^. Inspired by nature, catalytic cascade reaction in chemical synthesis has been developed to enable multistep transformation in a one-pot manner, thereby circumventing the isolation of unstable or toxic intermediates and improving the atom economy and overall yields^6,7^. In cascade catalysis, the reactivity and selectivity could additionally be enhanced by evading equilibrium reactions by the cooperative effects of multiple catalysts or multiple catalytic active sites^6,7^. But how to achieve satisfactory cascade catalysis remains challenging in terms of selectivity. The reaction tends to stay in the intermediate without subsequent conversion since the cascade steps are hard to be coupled in a precise and smooth relay manner. This work proposes a strategy on cascade synergistic catalysis for the efficient selective oxidation of glycerol to glyceric acid (GLYA) as a showcase, which is a typical cascade oxidation process and has been a longtime key problem towards the selective oxidation of C-O bonds in the biomass value-added processes.

Glycerol is an abundant side-product in the production of biodiesel via transesterification, which has been of major concern^8,9^. The selective oxidation of glycerol to GLYA, which possesses physiological activity and is used directly or as intermediates in the fields of drug and pharmaceutical manufacture, is an important value-added reaction from polyols^10–12^. With the rising global concerns about energy and the environment, the exploitation of biomass as clean and renewable energy provides one promising strategy to conform the demand of escalating energy^13–15^. The efficient conversion of glycerol to value-added fine chemicals is quite demanded more than ever^10–12,16–22^. The cascade catalytic process includes the dehydrogenation of primary O-H and C-H bonds of glycerol to glyceraldehyde (GLAD) as intermediate, and the subsequent C=O activation of GLAD followed by the O-H insertion and the additional dehydrogenation to GLYA^23–25^. The problem of low GLYA selectivity, the common challenge in cascade catalysis, is one of the main challenges due to the powerless transformation from GLAD to GLYA, and/or the deep oxidation and C-C cleavage. The other challenge has been that the maximization of the dehydrogenation of primary C-H bonds of glycerol, which is recognized as the rate-determining step (RDS)^23–25^, to achieve high activity is also difficult. Considerable efforts have been focused on the exploration of heterogeneous catalysts, including supported Pt^23–26^, and Pt-based bimetallic alloy (i.e., Au^27–29^, Zn^30^, Sn^31^, Cu^32,33^, or Co^34,35^) catalysts. Despite that alloying Pt with a second metal component^27–35^ provides a significant promotion of the dehydrogenation of C-H bonds of glycerol, the sole PtM catalytic active site could not work well to simultaneously achieve high GLYA selectivity at such high conversion, only obtaining a moderate GLYA yield.

**Fig. 1:**
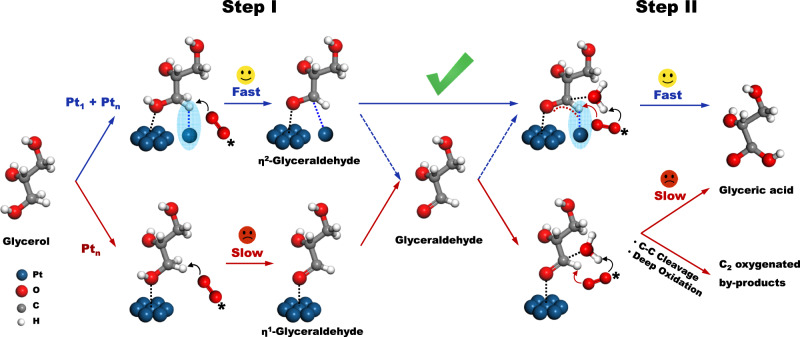
Proposed cascade synergistic catalysis strategy by atomic Pt_1_ and cluster Pt_n_ sites in the selective oxidation from glycerol to GLYA as a showcase. Atomic Pt_1_ site provides an enhanced C-H activation (marked in blue shade) in the cascade synergy, forming the η^2^-adsorbed GLAD.

Herein, a cascade synergistic catalysis strategy on enhanced C-H activation by atomic Pt_1_ synergistic with low-coordinated Pt-Pt for the cascade selective oxidation of glycerol to GLYA has been demonstrated. Supported multiple PtCu active sites, integrating atomic Pt_1_ and cluster Pt_n_ on well-defined Cu-CuZrO_x_, have been developed to demonstrate the cascade synergistic catalysis strategy. Our rationale is motivated by the literature survey^36,37^ on Pt/Cu single-atom alloy in catalytic dehydrogenation, in which the isolated Pt atoms with unique electron-rich characters could facilitate the activation of the C-H bonds with intermediate barriers. In this work (Fig. 1), the proposed cascade synergistic catalysis includes: (1) the enhanced C-H activation on atomic Pt_1_ and O-H activation on cluster Pt_n_ in the oxidation of glycerol to η^2^-adsorbed GLAD (Step I); (2) the O-H insertion of η^2^-adsorbed GLAD followed by enhanced C-H activation on atomic Pt_1_ in the tandem oxidation of GLAD to GLYA (Step II). Superior to the sole cluster/nanoparticles PtM catalysis, the enhanced C-H activation by the atomic Pt_1_ in the cascade synergy has been proposed not only to promote the glycerol conversion to GLAD, but to enhance the transformation of GLAD to GLYA in high selectivity to avoid deep oxidation and C-C cleavage, accordingly achieving both high glycerol conversion and high GLYA selectivity.

## Results

### Atomic and cluster Pt sites on Cu-CuZrO_x_

To be specific, the introduction of atomic Pt and cluster Pt sites was achieved by a galvanic replacement reaction on the surface of Cu nanoparticles (Supplementary Fig. 1a), which were prepared by in situ dissolution from the zirconium/copper mixed oxide. According to the ratio of zirconium and copper of 4.3: 1 from ICP characterization, the precursor is denoted as Cu_1_Zr_4.3_O_9.6_. As shown in the XRD pattern (Supplementary Fig. 1b), Cu_1_Zr_4.3_O_9.6_ was identified as CuZrO_3_ phase in orthorhombic shape (JCPDS 43–0953) with no other impurities. The morphology is an agglomerated flakes type with a rough surface (Supplementary Fig. 2a). In the H_2_-TPR (Supplementary Fig. 2b), two observed H_2_ consumptions at 176 and 233 ^o^C could be attributed to the reduction of Cu-O-Cu and Cu-O-Zr species^38^, respectively. The reduction was carried out under 450 ^o^C with an H_2_ stream to obtain supported Cu nanoparticles with a partial Cu reduction from bulk Cu_1_Zr_4.3_O_9.6_ (Cu-CuZrO_x_), according to the Cu 2*p* core-level X-Ray photoelectron spectroscopy (XPS) spectra (Supplementary Fig. 3a). As shown in the HRTEM images (Supplementary Fig. 4a), Cu nanoparticles with a maximum size distribution of around 3.4 nm are well-dispersed on the agglomerated oxide flakes in particle size of 50–100 nm. The Cu (111) plane (lattice spacing: 0.209 nm) is resolved (Supplementary Fig. 4b), consistent with the appearance of a tiny reflection of the Cu (111) in the XRD pattern (Supplementary Fig. 1b).

**Fig. 2:**
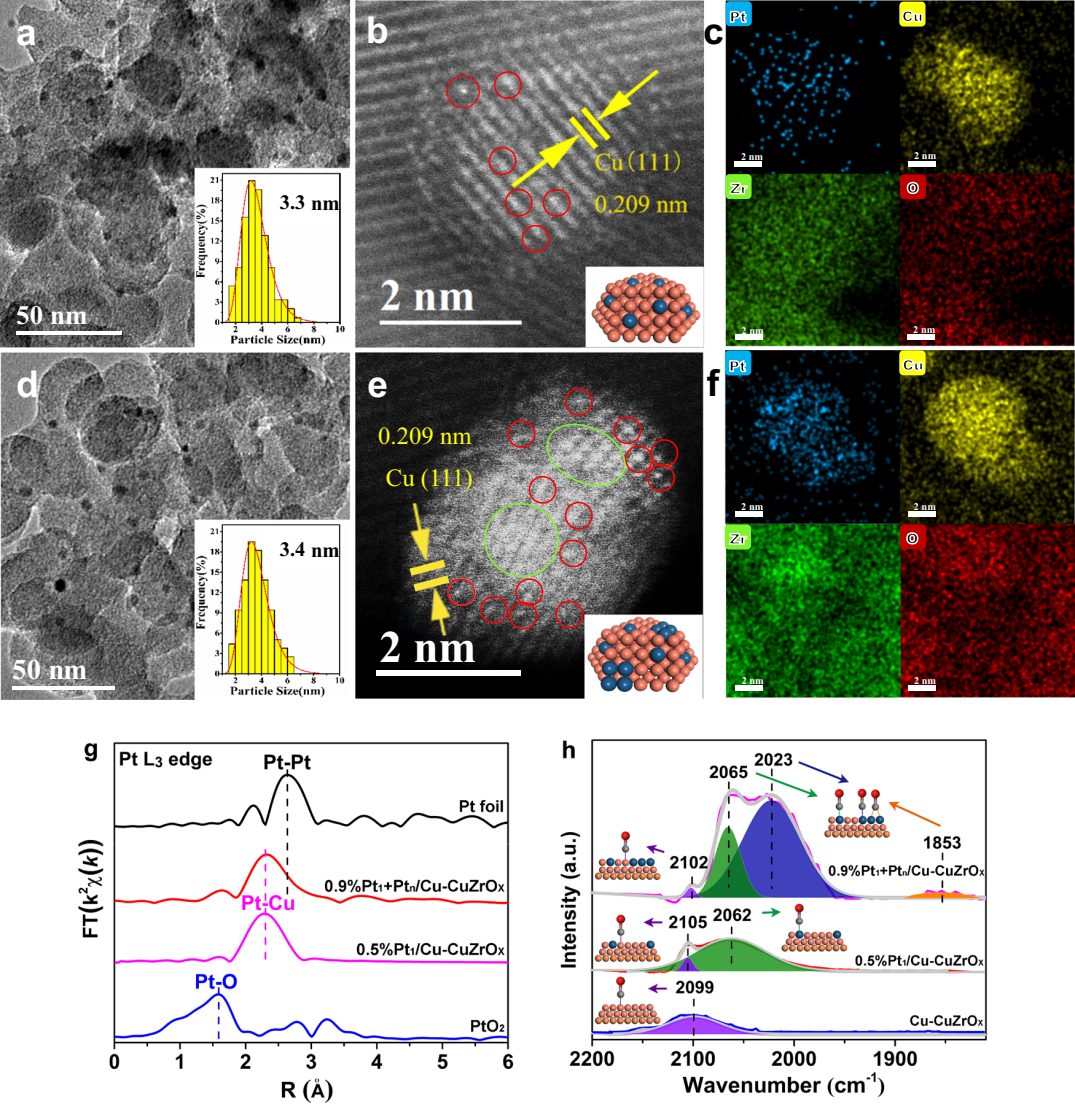
Pt dispersion state and coordination configuration. **a–f** Representative HRTEM images (**a**, **d**), AC-HAADF-STEM images (**b**, **e**), and HADDF-STEM-EDS element mappings (**c**, **f**) of 0.5%Pt_1_/Cu-CuZrO_x_ (**a–c**) and 0.9%Pt_1_+Pt_n_/Cu-CuZrO_x_ (**d–f**). **g** Pt L3-edge FT-EXAFS spectra of 0.5%Pt_1_/Cu-CuZrO_x_, and 0.9%Pt_1_+Pt_n_/Cu-CuZrO_x_ with bulk Pt foil and PtO_2_ as references. **h** CO-DRIFTS spectra of Cu-CuZrO_x_, 0.5%Pt_1_/Cu-CuZrO_x_, and 0.9%Pt_1_+Pt_n_/Cu-CuZrO_x_.

Afterward, a galvanic replacement method was employed to introduce Pt atoms onto the Cu surface with a Pt loading of 0.5 and 0.9 wt% (Supplementary Table 1). It is noted from XRD patterns that the Pt replacement made no significant changes in the crystalline structure without resolved Pt phase (Supplementary Fig. 1b), implying a high dispersion degree of Pt species. In a Pt loading of 0.5 wt% (Fig. 2a), the nanoparticles in a Cu (111) plane show a maximum size distribution of around 3.3 nm on the oxide flakes close to Cu-CuZrO_x_ (Supplementary Fig. 4a). In the aberration-corrected high-angle annular darkfield scanning transmission electron microscopy (AC-HAADF-STEM) image (Fig. 2b), single Pt atoms (red circle) are located on Cu (111) planes and no Pt atoms are resolved on the oxide. The STEM-coupled energy dispersive spectroscopy (EDS) element mapping (Fig. 2c) of Pt (blue), Cu (yellow), Zr (olive), and O (red) presents a uniform and high dispersion of Pt sites on the Cu nanoparticles, confirming the atomically-dispersed Pt site (0.5%Pt_1_/Cu-CuZrO_x_). With an increased Pt loading of 0.9%, no visible Pt nanoparticles are resolved instead of Cu (111) nanoparticles (~3.4 nm) (Fig. 2d). Demonstrated by AC-HAADF-STEM images (Fig. 2e), a few Pt clusters around 1.2 nm (olive circle) coexist nearby the atomic Pt (red circle) (0.9%Pt_1_ + Pt_n_/Cu-CuZrO_x_). The 3D surface simulation of the marked regions (yellow rectangles) verifies the adjacent spatial distribution of atomic Pt_1_ and cluster Pt_n_ (Supplementary Fig. 5). In the brightness intensity profiles (Supplementary Fig. 6), a distance between atomic Pt_1_ and the Pt_n_ cluster for the two marked line I and II is determined to be ~0.18 and ~0.22 nm, further confirming the adjacent spatial distribution. Considering the bond length (1.439 Å) of the primary C-O in glycerol, it provides the possibility to form synergistic adsorption of the primary C-O on the adjacent atomic Pt_1_ and cluster Pt_n_ sites as expected. The STEM-EDS mappings (Fig. 2f) confirm the coexistence of atomic Pt and cluster Pt sites. Additional HRTEM and AC-HAADF-STEM images are provided in Supplementary Fig. 7.

In the Fourier-transformed extended X-ray absorption fine structure spectra (FT-EXAFS, Fig. 2g) at the Pt L3-edge, 0.5%Pt_1_/Cu-CuZrO_x_ exhibits one dominant peak in the region around 2.3 Å located between PtO_2_ and Pt foil, demonstrating the formation of Pt-Cu coordination according to the references^36,37^. As fitted (Supplementary Table 2), only 8.3 Pt-Cu coordination was identified without any Pt-Pt or Pt-O coordination detected. Whereas, 0.9%Pt_1_ + Pt_n_/Cu-CuZrO_x_ presents a much broader peak in the region around 2.3 Å with an asymmetric shoulder peak in the region around 2.6 Å, corresponding to Pt-Cu and Pt-Pt coordination structure with fitted 6.1 Pt-Cu and 3.0 Pt-Pt coordination, confirming the coexistence of atomic Pt_1_ and cluster Pt_n_. In situ CO-absorbed diffuse reflectance infrared Fourier transform spectroscopy measurements (CO-DRIFTS, Fig. 2h) were performed to probe the atomic geometry configuration of Pt sites on the Cu surface. On Cu-CuZrO_x_ as control, a weak and broad absorption band at 2099 cm^−1^ associated^39,40^ with chemisorbed CO on Cu^0^ species appears. On 0.5%Pt_1_/Cu-CuZrO_x_, two deconvoluted bands by multi-peaks Gaussian fitting are observed at 2105 and 2062 cm^−1^, respectively. By comparison to Cu-CuZrO_x_, the former band is attributed^39,40^ to the chemisorbed CO absorption on Cu^0^ species in an electron-deficient state due to the Cu-Pt coordination. The latter is associated^41–43^ with the linearly-bonded CO on atomic Pt. On 0.9%Pt_1_ + Pt_n_/Cu-CuZrO_x_, besides the bands for CO adsorbed on Cu^0^ species (2102 cm^−1^) and CO linearly-adsorbed on atomic Pt (2065 cm^−1^), two bands at 2023 and 1853 cm^−1^ attributed^42,43^ to CO linearly- and bridged-adsorbed on Pt clusters are resolved, respectively.

**Fig. 3:**
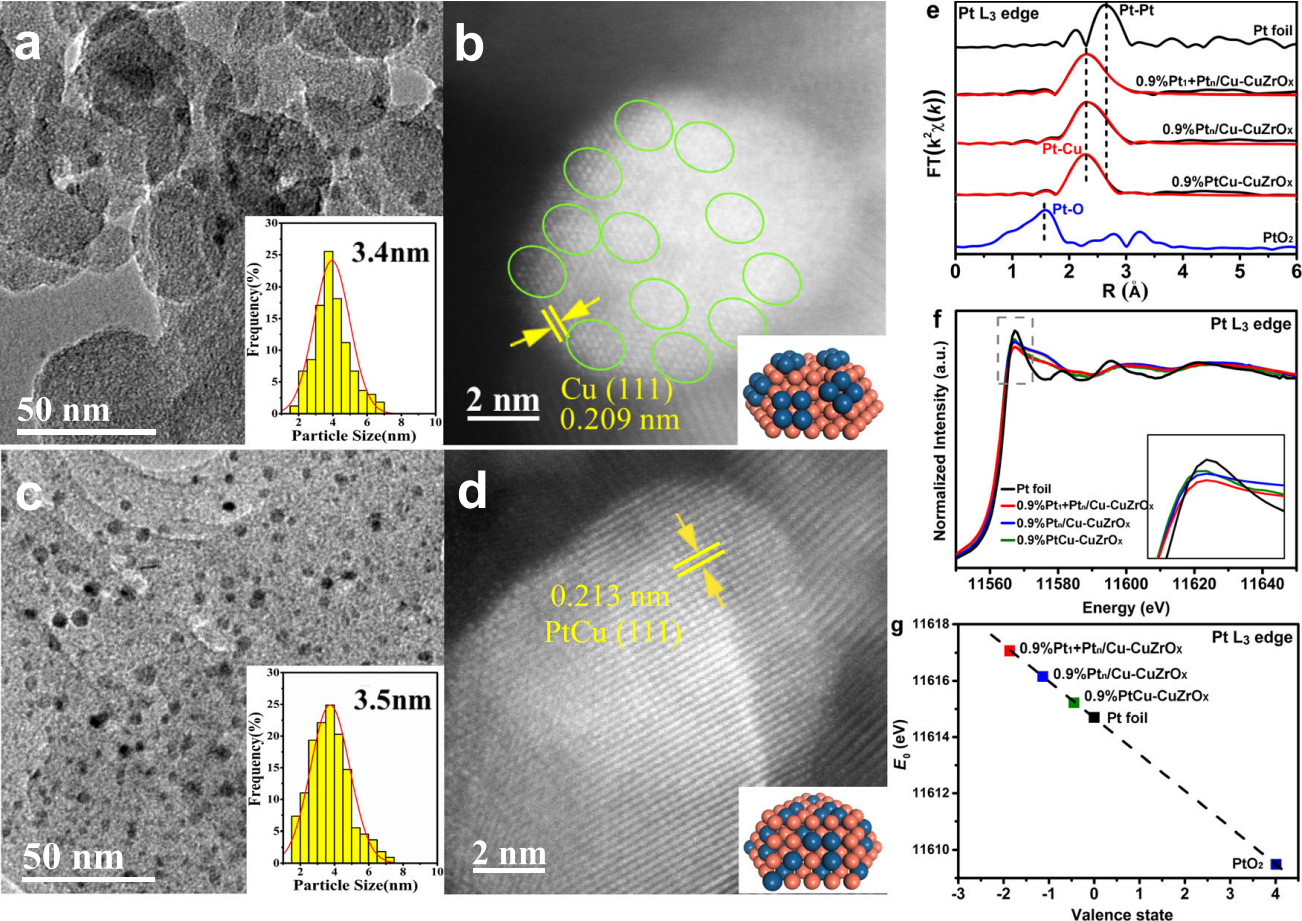
Modulation on Pt coordination configuration and electronic state. **a–d** Representative HRTEM and AC-HAADF-STEM images of 0.9%Pt_n_/Cu-CuZrO_x_ (**a**, **b**) and 0.9%PtCu-CuZrO_x_ (**c**, **d**). **e–g** Pt L3-edge FT-EXAFS spectra (**e**), normalized XANES spectra (**f**), and the valence state analysis (**g**) of 0.9%Pt_1_+Pt_n_/Cu-CuZrO_x_, 0.9%Pt_n_/Cu-CuZrO_x_, and 0.9%PtCu-CuZrO_x_ with bulk Pt foil and PtO_2_ as references.

Additional PtCu bimetallic catalysts possessing only Pt clusters (0.9%Pt_n_/Cu-CuZrO_x_) and unique bulk PtCu alloy nanoparticles (0.9%PtCu-CuZrO_x_) were designed. 0.9%Pt_n_/Cu-CuZrO_x_ and 0.9%PtCu-CuZrO_x_ were prepared by incipient wetness impregnation to introduce PtCl_6_^2-^ onto Cu-CuZrO_x_ or CuZrO_x_ followed by reduction. For 0.9%Pt_n_/Cu-CuZrO_x_, no visible Pt nanoparticles are resolved instead of Cu nanoparticles in a particles size of 3.4 nm (Fig. 3a). Pt clusters around 1.2 nm (olive circle) are observed on Cu (111) planes without visible atomic Pt, other than on the oxide surface (Fig. 3b). For 0.9%PtCu-CuZrO_x_, PtCu alloy (111) nanoparticles with a particle size of 3.5 nm were obtained (Fig. 3c), and no visible individual Pt atoms are distinguished in the AC-HAADF-STEM image (Fig. 3d). In R-spaced FT-EXAFS spectra (Fig. 3e), besides Pt-Cu coordination (2.3 Å), 0.9%Pt_n_/Cu-CuZrO_x_ possesses much more Pt-Pt coordination than 0.9%Pt_1_ + Pt_n_/Cu-CuZrO_x_ in the light of the larger asymmetric shoulder peak in the region around 2.6 Å. 0.9%PtCu-CuZrO_x_ exhibits one dominant peak (2.3 Å) with a tiny shoulder peak (2.6 Å), indicating Pt-Cu as the main Pt coordination with a small amount of Pt-Pt coordination. Moreover, no resolved PtO coordination is observed. As a comparison for 0.9%Pt_1_ + Pt_n_/Cu-CuZrO_x_ in 6.1 Pt-Cu and 3.0 Pt-Pt coordination, 4.1 Pt-Cu and 2.9 Pt-Pt coordination are identified for 0.9%Pt_n_/Cu-CuZrO_x_, and 4.1 Pt-Cu and 2.3 Pt-Pt coordination for 0.9%PtCu-CuZrO_x_, respectively (Supplementary Table 2). CO-DRIFTS spectra of the physically-mixed 0.5%Pt_1_/Cu-CuZrO_x_ and 0.9%Pt_n_/Cu-CuZrO_x_ were collected as control (Supplementary Fig. 8). The CO linearly-adsorbed bands on Pt_1_ (2062 cm^−1^) and cluster Pt_n_ (2033 cm^−1^) on the physically-mixed 0.5%Pt_1_/Cu-CuZrO_x_ and 0.9%Pt_n_/Cu-CuZrO_x_ shift to 2065 and 2023 cm^−1^ on 0.9%Pt_1_ + Pt_n_/Cu-CuZrO_x_ with an increased discrepancy (Fig. 2h), which could be caused by the CO molecule conjugation^44^ on adjacent atomic and cluster Pt in 0.9%Pt_1_ + Pt_n_/Cu-CuZrO_x_.

In the Pt L3-edge spectra of X-ray absorption near-edge structure spectra (XANES, Fig. 3f), each PtCu displays a white line intensity below Pt foil, indicative of electron-rich Pt (Pt^δ-^). As enlarged in the inset, with the reference of Pt foil, the intensity of the red line indicates 0.9%Pt_1_ + Pt_n_/Cu-CuZrO_x_ is the most negative-charged, and subsequently, 0.9%Pt_n_/Cu-CuZrO_x_ and 0.9%PtCu-CuZrO_x_. In the first-derivative of absorption edge in normalized XANES spectra, the absorption threshold (*E*_0_) was obtained (Supplementary Fig. 9), which was then plotted as a function of the oxidation state (Fig. 3g). The Pt^δ-^ valence state has been quantified in a δ value of 1.85, 1.13, and 0.44 for 0.9%Pt_1_ + Pt_n_/Cu-CuZrO_x_, 0.9%Pt_n_/Cu-CuZrO_x_, and 0.9%PtCu-CuZrO_x_ respectively from the linear fitting result in light of the PtO_2_ and Pt foil. In Cu 2*p*3/2 XPS spectra (Supplementary Fig. 3a), the peak assigned^38^ to Cu^0^ or Cu^I^ species shifts to lower binding energy after Pt loading, indicating an electron donation from Cu to Pt atoms. In the Cu X-ray excited Auger electron spectroscopy (XAES) spectra (Supplementary Fig. 3b), the kinetic energy of the peak assigned^45^ to Cu^0^ species shifts to low energy compared to Cu-CuZrO_x_, confirming the electron-deficient state for Cu in the Pt-Cu bonds, in accordance with the XANES spectra analysis (Fig. 3f, g).

### Cascade synergistic activation by atomic Pt_1_ and cluster Pt_n_ sites

**Fig. 4:**
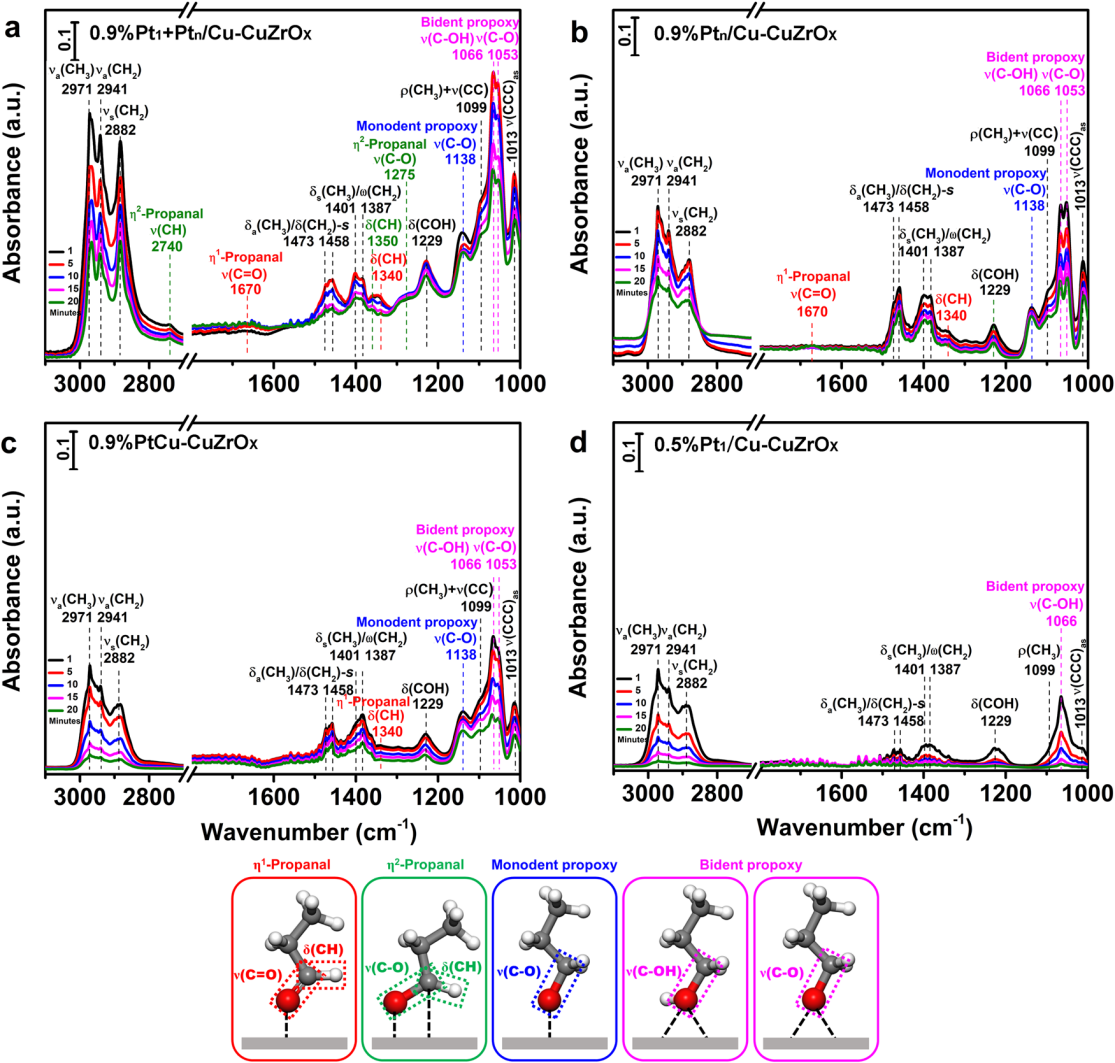
The activation of the primary O-H and C-H bonds. In situ FT-IR spectra after the adsorption of 1-propanol for 30 min followed by desorption at desorption times of 1, 5, 10, 15, and 20 min (from top to bottom) on 0.9%Pt_1_+Pt_n_/Cu-CuZrO_x_ (**a**), 0.9%Pt_n_/Cu-CuZrO_x_ (**b**), 0.9%PtCu-CuZrO_x_ (**c**), and 0.5%Pt_1_/Cu-CuZrO_x_ (**d**). The simulated adsorption models are displayed corresponding to the absorption band labeled in the same color.

The activation of glycerol has been first investigated by in situ FT-IR spectra after the adsorption of 1-propanol for 30 min followed by desorption, which possesses only primary O-H bonds (Fig. 4). On 0.9%Pt_1_ + Pt_n_/Cu-CuZrO_x_ (Fig. 4a and Supplementary Table 3), besides the bands attributed^46–48^ to adsorbed bidentate 1-propanol and propoxy (marked in magenta), adsorbed monodentate propoxy (marked in blue), and η^1^-adsorbed propanal (marked in red), the bands at 2740, 1275, and 1350 cm^−1^ assigned^46–48^ to *ν*(CH), *ν*(C-O) and δ(CH) of η^2^-adsorbed propanal (marked in olive) appear. The corresponding adsorption models labeled in the same color are also displayed below. As the desorption time increased, the bands gradually decrease except for the η^2^-adsorbed mode, which is inferred to be stable in such a synergistic activation. While on 0.9%Pt_n_/Cu-CuZrO_x_ (Fig. 4b) or 0.9%PtCu-CuZrO_x_ (Fig. 4c), similar absorption bands with weakened intensity for adsorbed 1-propanol, propoxy species, and η^1^-adsorbed propanal appear, except that no bands for η^2^-adsorbed propanal are resolved. Combined with the electronic state analysis, Pt^δ-^-Cu bonds are responsible for the dehydrogenation of the C-H bonds as expected and the increasing electron-rich state in 0.9%Pt_1_ + Pt_n_/Cu-CuZrO_x_ promotes the activation of the C-H bonds. Moreover, by comparing the area ratio of *ν*(C-O) and *ν*(C-OH) of the adsorbed bidentate 1-propanol (marked in magenta), 0.9%Pt_n_/Cu-CuZrO_x_ exhibits superior O-H activation beyond 0.9%Pt_1_ + Pt_n_/Cu-CuZrO_x_ and 0.9%PtCu-CuZrO_x_, confirming that the Pt-Pt bonds are responsible for the dehydrogenation of the primary O-H bonds to propoxy and more Pt-Pt coordination (Supplementary Table 2) is preferred. As a control, on 0.5%Pt_1_/Cu-CuZrO_x_ (Fig. 4d), almost no propoxy and propanal species were observed except for the adsorbed non-dissociated 1-propanol species.

**Fig. 5:**
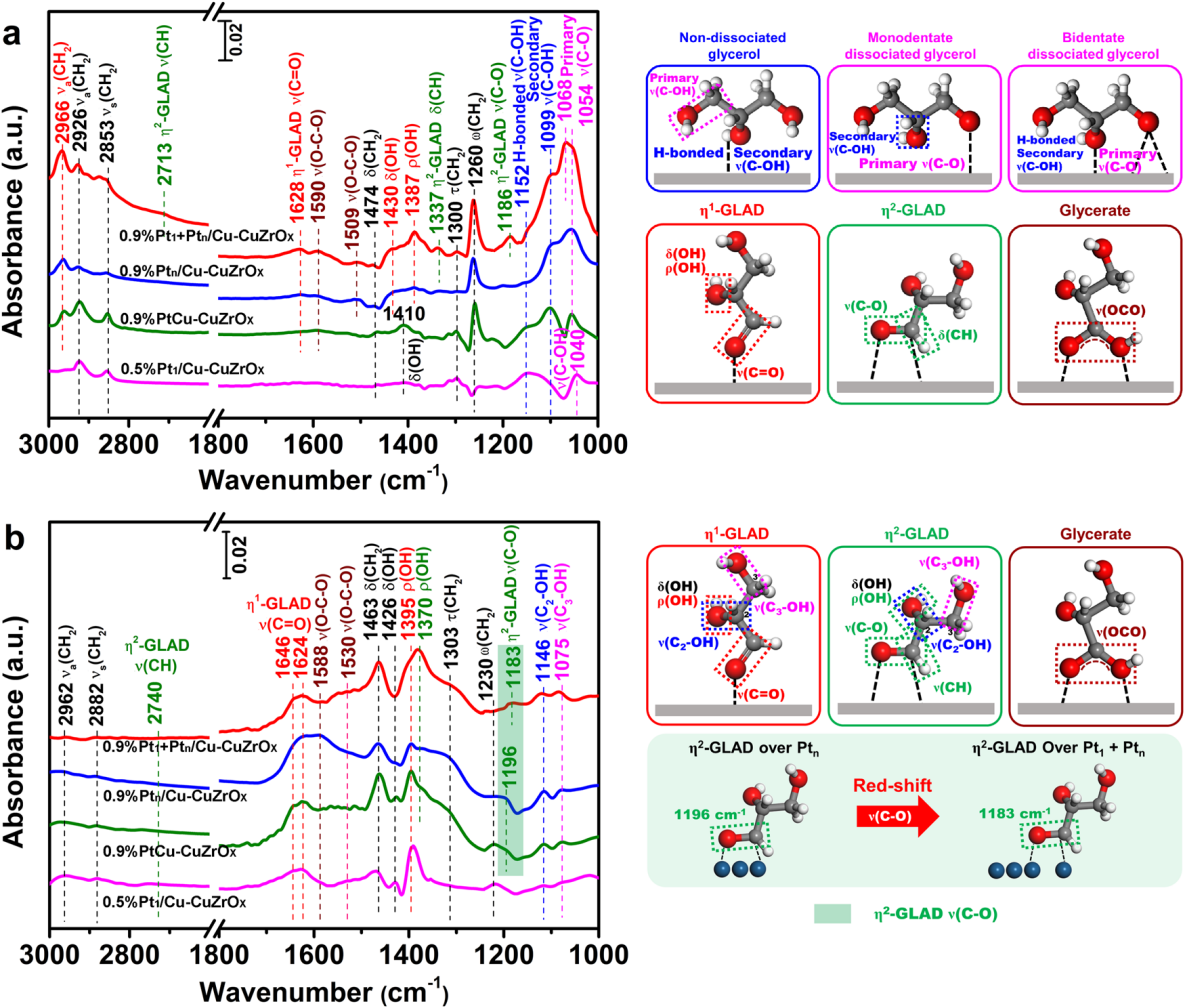
The adsorption and the activation of glycerol and glyceraldehyde. In situ FT-IR spectra of the adsorption of glycerol (**a**) and glyceraldehyde (**b**) on 0.9%Pt_1_+Pt_n_/Cu-CuZrO_x_, 0.9%Pt_n_/Cu-CuZrO_x_, 0.9%PtCu-CuZrO_x_, and 0.5%Pt_1_/Cu-CuZrO_x_ with the simulated adsorption models corresponding to the absorption bands labeled in the same color. Schematic illustrations of glyceraldehyde adsorption in η^2^-GLAD mode over Pt_1_ + Pt_n_ sites and Pt_n_ sites are also displayed.

Then the glycerol activation on atomic Pt_1_ and cluster Pt_n_ sites has been investigated (Fig. 5a and Supplementary Table 4). Taking pristine glycerol as reference (Supplementary Fig. 10a), on 0.9%Pt_1_ + Pt_n_/Cu-CuZrO_x_ two strong absorption bands at 1068 and 1054 cm^−1^ attributed^49–52^ to primary *ν*(C-O) of bidentate and monodentate dissociated glycerol (marked in magenta), a strong band at 1099 cm^−1^ to^49–52^ the secondary *ν*(C-OH) of non-dissociated glycerol (marked in blue), and a tiny band at 1152 cm^−1^ to^49–52^ non-dissociated glycerol with secondary O-H group forming hydrogen bonds on the metal oxides (marked in blue) appear, indicating the dominance on the dissociated activation of the primary O-H bands. Besides η^1^-adsorbed GLAD (marked in red), the bands at 2713, 1337, and 1186 cm^−1^ assigned^53,54^ to *ν*(CH), δ(CH), and *ν*(C-O) of η^2^-adsorbed GLAD (marked in olive) appear. No visible activation of the secondary C-H bonds to dihydroxyacetone^51^ has been detected. While on 0.9%Pt_n_/Cu-CuZrO_x_ or 0.9%PtCu-CuZrO_x_, no obvious bands for η^2^-adsorbed GLAD are resolved, and only weakened primary O-H and C-H bond activation are detected. More H-bonded secondary *ν*(C-OH) species on 0.9%PtCu-CuZrO_x_ are observed. As a control, on 0.5%Pt_1_/Cu-CuZrO_x_, only H-bonded secondary *ν*(C-OH) species appear without any dissociated GLAD detected. Therefore, the dominant activation of primary O-H and C-H bonds of glycerol in η^2^-GLAD mode on atomic Pt_1_ and cluster Pt_n_ sites has been identified, consistent with the in situ FT-IR spectra of the 1-propanol adsorption. In addition, the appearance of *ν*(OCO) in glycerate (marked in wine) could be attributed^51^ to the adsorption of formed C=O on metal and the metal oxides.

**Fig. 6:**
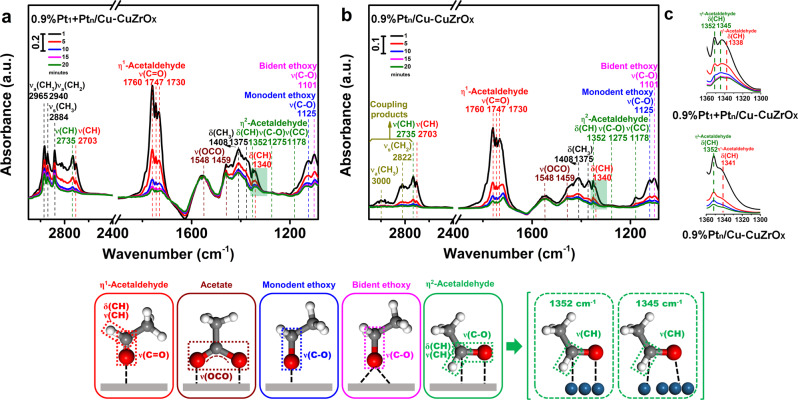
The activation of the C=O bonds in aldehyde. In situ FT-IR spectra after the adsorption of acetaldehyde for 30 min followed by desorption at desorption times of 1, 5, 10, 15, and 20 min (from top to bottom) on 0.9%Pt_1_+Pt_n_/Cu-CuZrO_x_ (**a**), and 0.9%Pt_n_/Cu-CuZrO_x_ (**b**), with enlarged spectra between 1360 and 1300 cm^−1^ filled in olive shading (**c**). The simulated adsorption models are displayed corresponding to the adsorption band labeled in the same color.

The activation of the aldehyde group has been first investigated by in situ FT-IR spectra of the adsorption of acetaldehyde (Fig. 6). On 0.9%Pt_1_ + Pt_n_/Cu-CuZrO_x_ (Fig. 6a and Supplementary Table 5), the bands at 1760, 1747 and 1730 cm^−1^ assigned^55–57^ to *ν*(C=O) of η^1^-adsorbed acetaldehyde (marked in red) and bands at 1275 and 1352 cm^−1^ assigned^55–57^ to *ν*(C-O) and δ(CH) of η^2^-adsorbed acetaldehyde (marked in olive) both appear. On 0.9%Pt_n_/Cu-CuZrO_x_ (Fig. 6b), similar absorption bands for η^1^- and η^2^-adsorbed acetaldehyde with sharply-weakened intensity are observed, except that a broad band around 3000 cm^−1^ associated^55^ with the coupling products of adsorbed acetaldehyde appears (marked in dark yellow). As the desorption time increased, besides the band at 1352 cm^−1^ assigned^55–57^ to δ(CH) in η^2^-acetaldehyde species on sole Pt_n_ sites, a band at 1345 cm^−1^ assigned to a weaker δ(CH) also appear, which could be generated by the stronger Pt-C interaction on more electron-deficient Pt_1_ sites in 0.9%Pt_1_ + Pt_n_/Cu-CuZrO_x_. It is envisaged that the generation of η^2^-adsorbed acetaldehyde on atomic Pt_1_ and cluster Pt_n_ sites make the C=O activation much faster and inhibits the side coupling reaction. In addition, the *ν*(OCO) bands attributed^55^ to the C=O adsorption on metal sites and oxygen of metal oxides (marked in wine) also appear. For GLAD activation (Fig. 5b), taking pristine GLAD as reference (Supplementary Fig. 10b), η^1^- (marked in red) and η^2^-adsorbed (marked in olive) GLAD species^53,54^ are both observed on 0.9%Pt_1_+Pt_n_/Cu-CuZrO_x_, 0.9%Pt_n_/Cu-CuZrO_x_, and 0.9%PtCu-CuZrO_x_. On 0.9%Pt_1_ + Pt_n_/Cu-CuZrO_x_, η^2^-adsorbed GLAD are dominant adsorbed species. While on 0.9%Pt_n_/Cu-CuZrO_x_ and 0.9%PtCu-CuZrO_x_, η^1^-adsorbed GLAD species appear as predominant adsorption. It is worth noting that the η^2^-GLAD *ν*(C-O) band (filled in olive) at 1196 cm^−1^ on 0.9%Pt_n_/Cu-CuZrO_x_ and 0.9%PtCu-CuZrO_x_ displays an obvious red-shift to 1183 cm^−1^ on 0.9%Pt_1_ + Pt_n_/Cu-CuZrO_x_ (Fig. 5b, inset), indicating a weaker *ν*(C-O) in the η^2^-GLAD on Pt_1_ and Pt_n_ sites than that on sole Pt_n_ sites, which could be attributed to the stronger Pt-C interaction on more electron-deficient Pt_1_ sites in 0.9%Pt_1_ + Pt_n_/Cu-CuZrO_x_, in accordance with the acetaldehyde adsorption. It provides experimental evidence on the formation of η^2^-adsorbed GLAD by the synergistic activation of atomic Pt_1_ and cluster Pt_n_ sites. More glycerate species (marked in wine) are observed on 0.9%Pt_n_/Cu-CuZrO_x_, inferring that η^1^-adsorbed GLAD species facilitate the formation of *ν*(OCO) adsorption on metal and oxides. For control, 0.5%Pt_1_/Cu-CuZrO_x_ has only η^1^-adsorbed GLAD species. In each case, no obvious C_2_-OH and C_3_-OH activation in GLAD is detected.

**Fig. 7:**
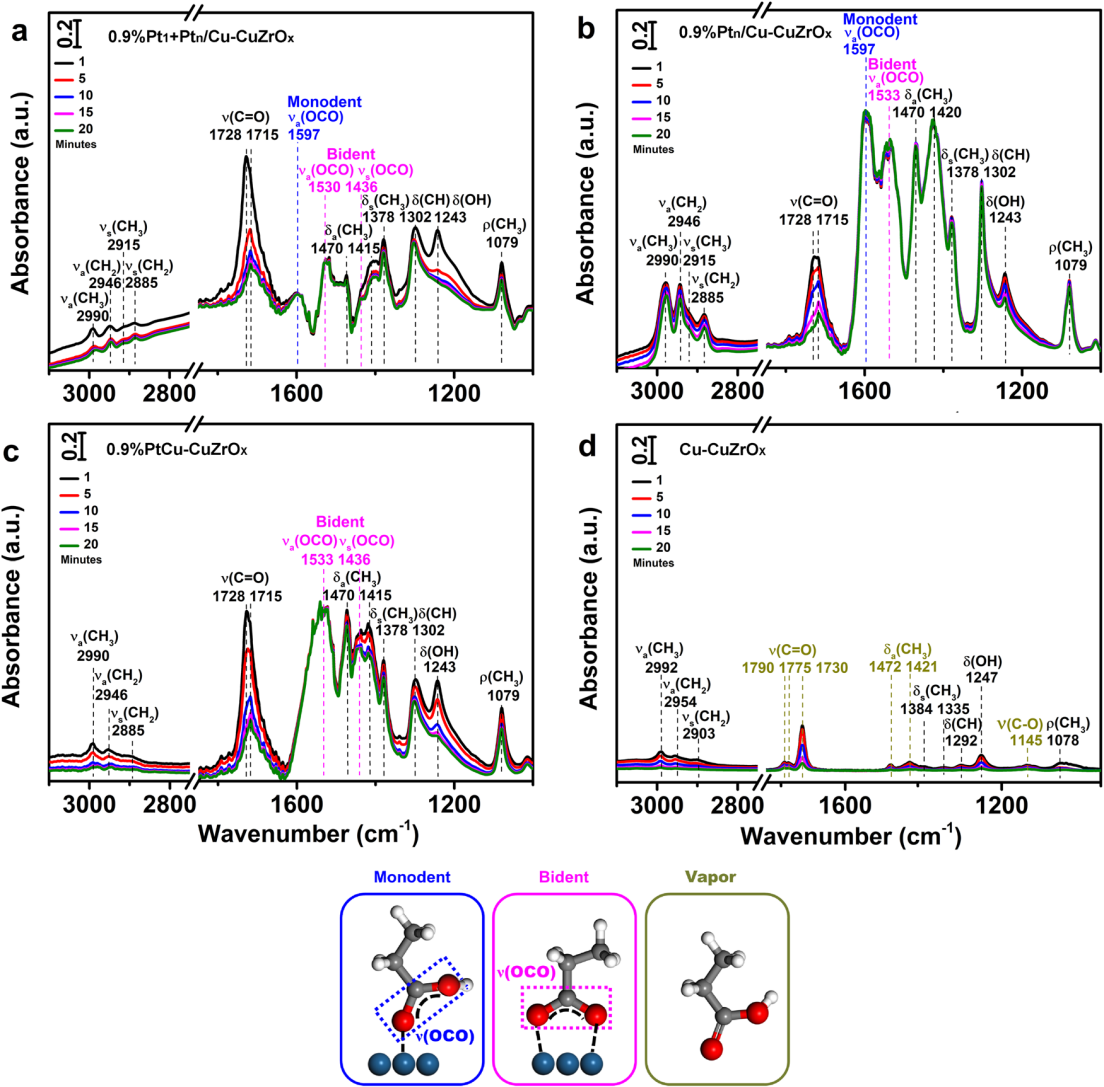
The desorption behavior of carboxyl groups. In situ FT-IR spectra after the adsorption of propionic acid for 30 min followed by desorption at desorption times of 1, 5, 10, 15, and 20 min (from top to bottom) on 0.9%Pt_1_+Pt_n_/Cu-CuZrO_x_ (**a**), 0.9%Pt_n_/Cu-CuZrO_x_ (**b**), 0.9%PtCu-CuZrO_x_ (**c**), and Cu-CuZrO_x_ (**d**) with simulated adsorption models displayed in the same color.

The desorption of carboxyl groups has been investigated by in situ FT-IR spectra after the adsorption of propionic acid followed by desorption (Fig. 7 and Supplementary Table 7). The adsorption of the carboxyl groups on the metal clusters or nanoparticles could result in monodentate and bidentate configuration considering the surface metal atom arrangement^58^. On 0.9%Pt_1_ + Pt_n_/Cu-CuZrO_x_ (Fig. 7a), a weak band at 1597 cm^−1^ identified^48,57,59^ as *ν*_a_(OCO) in monodentate configuration (marked in blue) and the bands at 1530 and 1436 cm^−1^ identified^48,57,59^ as *ν*_a_(OCO) and *ν*_s_(OCO) in bridging bidentate configuration (marked in magenta) on cluster Pt_n_ appear. On 0.9%Pt_n_/Cu-CuZrO_x_ (Fig. 7b), intensified *ν*_a_(OCO) absorption bands at 1597 and 1533 cm^−1^ in both monodentate and bridging bidentate adsorption on cluster Pt_n_ are identified, demonstrating strong adsorption of the carboxyl groups. On 0.9%PtCu-CuZrO_x_ (Fig. 7c), only *ν*_a_(OCO) absorption band in bridging bidentate adsorption on cluster Pt_n_ is observed. 0.9%Pt_1_ + Pt_n_/Cu-CuZrO_x_ with less Pt-Pt coordination quantity in the similar Pt-Pt coordination number with 0.9%Pt_n_/Cu-CuZrO_x_ exhibits better desorption of carboxyl groups. As a control, on pristine Cu-CuZrO_x_ (Fig. 7d), only tiny absorption bands assigned^58^ to the vapor propionic acid were detected. Combined with the GLAD adsorption (Fig. 5b), it is concluded that 0.9%Pt_1_ + Pt_n_/Cu-CuZrO_x_ exhibits both better activation of the aldehyde group and desorption of the carboxy group originated from the synergistic catalysis by atomic Pt_1_ and cluster Pt_n_ sites, which is envisaged to facilitate the direct transformation from GLAD to GLYA.

### Cascade synergistic catalysis during in situ surface reaction

**Fig. 8:**
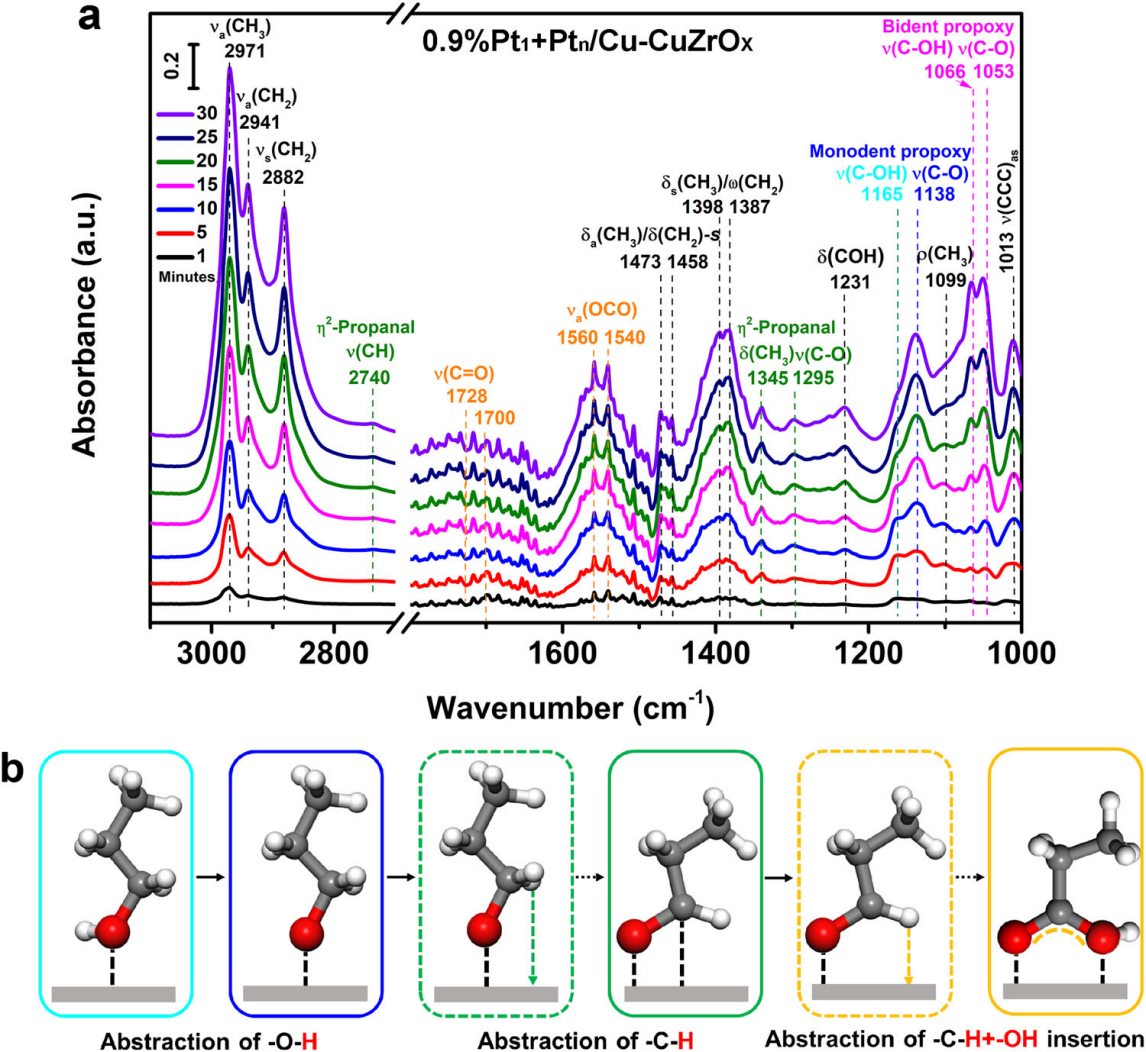
Schematic diagram of the reaction pathway during in situ surface reaction. **a** In situ time-resolved FT-IR spectra of 0.9%Pt_1_+Pt_n_/Cu-CuZrO_x_ on exposure to 1-propanol at 60 ^o^C was recorded in the presence of O_2_ and H_2_O. From bottom to top: 1, 5, 10, 15, 20, 25, and 30 min. Adsorbates were introduced to the chamber by bubbling the 1-propanol aqueous solution with O_2_/Ar (v:v = 1:5) flow (40 mL/min). **b** Proposed surface reaction process on the oxidation of 1-propanol to propionic acid.

In situ time-resolved FT-IR spectra of 0.9%Pt_1_ + Pt_n_/Cu-CuZrO_x_ on exposure to 1-propanol at 60 ^o^C in a flow of O_2_ and H_2_O were recorded to further elucidate the cascade synergistic catalysis and the evolution of surface adsorbed intermediates in the oxidation of glycerol to GLYA (Fig. 8a). The bands between 1600~1500 cm^−1^ assigned to the *ν*(OCO) of the adsorbed propionic acid in bridging bidentate configuration appear and gradually increase with on-steam time, accompanied with the appearance of *ν*(C=O) band of carboxyl groups since 15 min. It indicates that 1-propanol could be converted to propionic acid over 0.9%Pt_1_ + Pt_n_/Cu-CuZrO_x_ in the presence of O_2_ and H_2_O. With on-steam time, the area ratio of *ν*(C-O) at 1138 cm^−1^ of the adsorbed monodentate 1-propanol (marked in blue) to *ν*(C-OH) at 1165 cm^−1^ of the adsorbed monodentate propoxy (marked in cyan) gradually increases, but that of the adsorbed monodentate 1-propanol are not so visible (marked in magenta). With on-steam time, the area ratio of the total monodentate *ν*(C-O) and *ν*(C-OH) (1138 and 1165 cm^−1^) to the total bidentate ones (1053 and 1066 cm^−1^) gradually decreases. It evidences that the formation of propionic acid is originated from the adsorbed monodentate 1-propanol. The *ν*(CH), δ(CH_3_), and *ν*(C-O) bands of η^2^-adsorbed propanal (marked in olive) are clearly resolved without η^1^-adsorbed propanal bonds, confirming the synergistic activation of atomic Pt_1_ and cluster Pt_n_ on the C-H and O-H bonds, and demonstrating the η^2^-adsorbed propanal as the intermediates in the subsequent oxidation process to propionic acid. More importantly, the area ratio of *ν*(C-O) in propoxy at 1138 cm^−1^ (marked in blue) to *ν*(C-O) in η^2^-mode propanal at 1295 cm^−1^ (marked in olive) remarkable increased with on-steam time, indicating that the synergistic activation pathway is O-H activation on the Pt_n_ cluster followed by the C-H activation on the atomic Pt_1_ sites. The schematic illustration of the possible surface reaction process has been displayed in Fig. 8b. In situ XANES spectroscopy, which is sensitive for measuring the chemical states of Pt and Cu, was performed to investigate their changes during the catalytic reaction (Supplementary Fig. 11). It is found that the introduction of glycerol on 0.9%Pt_1_ + Pt_n_/Cu-CuZrO_x_ leads to a distinct low-energy shift of the white line peak in Pt L3-edge XANES spectra (Supplementary Fig. 11a). Then the exposure to O_2_ make the white line gradually return to the similar level of the fresh catalyst. As for the Cu K-edge XANES spectra after the sequential exposure to glycerol solution and O_2_ flow (Supplementary Fig. 11b), no visible change of the Cu adsorption edge has been observed, implying that no obvious effect on surface Cu species has been detected in the oxidation in this work. Cu species has been inferred to play a role in the electron donation to Pt atom to form Pt^δ-^-Cu coordination promoting the C-H activation of glycerol during the oxidation.

The selective oxidation of glycerol on 0.9%Pt_1_ + Pt_n_/Cu-CuZrO_x_ using ^18^O_2_ and H_2_^18^O were performed, respectively, to identify the reactive oxygen species (Supplementary Fig. 12), which were reported to participate in the oxidation process. With ^18^O_2_ labeling, ^18^O was not observed in the products of glycerol oxidation (Supplementary Fig. 12a). With H_2_^18^O labeling, the mass spectrum shows that one or two ^18^O atoms were incorporated into GLYA as the main product with a small amount of GLAD or DHA as by-products (Supplementary Fig. 12b). Combining with the previous reports^29,60^, it could be concluded that activated O_2_ and H_2_O were incorporated to form peroxide (OOH) and OH to abstract adsorbed C-H and O-H bonds, finally generating H_2_O_2_ and H_2_O (Supplementary Fig. 12c). To verify the H_2_O_2_ generation, the fresh reaction solution and reference samples were mixed with a mixed solution (P) containing phosphate buffer, *N*,*N*-diethylbenzene-1,4-diamine sulfate (DPD), and horseradish peroxidase (POD)^61^ to observe the change in color (Supplementary Fig. 12d). The solution color for the fresh reaction solution with P solution is visibly darker than that for each individual product with P, confirming the H_2_O_2_ establishment in the glycerol oxidation.

### Atomic Pt_1_ enhanced C-H activation synergistic with cluster Pt_n_ catalysis to promote glycerol conversion and GLYA selectivity

**Fig. 9:**
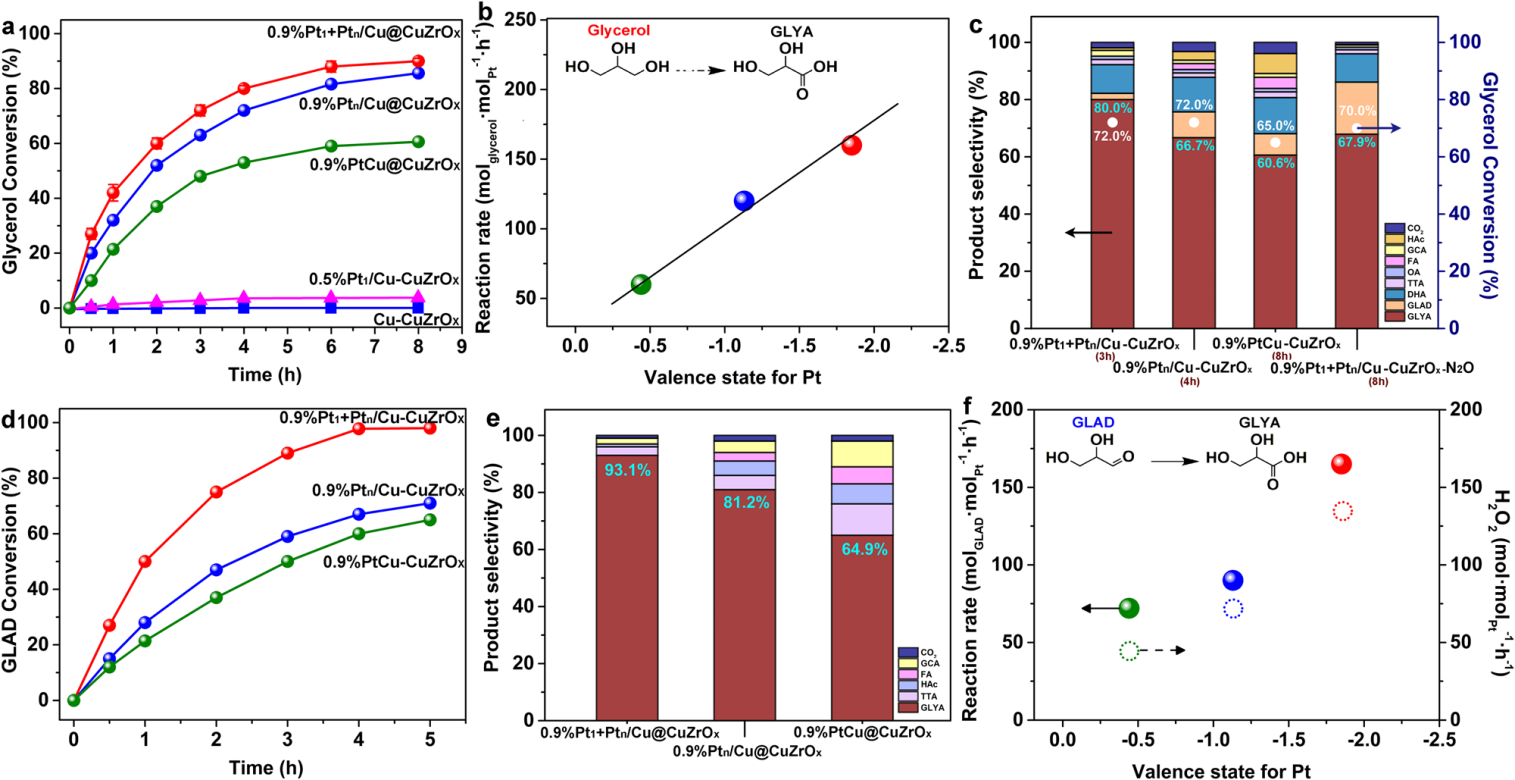
Pt electronic structure and the catalytic evaluation. **a–c** In the selective oxidation of glycerol, time-dependent glycerol conversion (**a**), profile of glycerol conversion rate with Pt valence state (**b**), and comparison of product selectivity at similar glycerol conversion (**c**). **d–f** In the selective oxidation of GLAD, time-dependent GLAD conversion (**d**), comparison of product selectivity (**e**), and profiles of GLAD conversion rate and H_2_O_2_ production rate with Pt valence state (**f**). Reaction conditions: ebullated bed, 15 mL of substrate aqueous solution (0.1 mol L^−1^), substrate/Pt (mol/mol) = 300, 60 ^o^C, O_2_ flow of 30 mL/min. Cu-CuZrO_x_ was input using the same mass with 0.9%Pt_1_ + Pt_n_/Cu-CuZrO_x_.

In the selective oxidation of glycerol in an ebullated bed with an O_2_ flow of 30 mL/min at 60 ^o^C, 0.9%Pt_1_ + Pt_n_/Cu-CuZrO_x_ catalyzed the conversion of glycerol to GLYA with a selectivity of 80.2 ± 0.2% at a glycerol conversion of 90.0 ± 0.1% in 8 h (Fig. 9 and Supplementary Table 8). Over 0.9%Pt_n_/Cu-CuZrO_x_ and 0.9%PtCu-CuZrO_x_, a GLYA selectivity of 66.9% at a glycerol conversion of 85.6%, and a GLYA selectivity of 60.6% at a glycerol conversion of 65.0% were achieved. While 0.5%Pt_1_/Cu-CuZrO_x_ only gives a very low conversion of 3.7% with a GLYA selectivity of 64.1% (Supplementary Fig. 13a) and do not change with time. As a control, the activity of Cu-CuZrO_x_ is negligible (0.4%) with oxalic acid (OA) and formic acid (FA) as main products (Supplementary Fig. 13b). It indicates that the cooperation of atomic Pt_1_ with cluster Pt_n_ promotes the glycerol conversion (Fig. 9a). In the profile of the reaction rate towards the Pt valence state (Fig. 9b), a good linear relationship between the activity and the Pt electron-rich state displays, further confirming the enhanced C-H activation by atomic Pt_1_ leads to promoted glycerol conversion. When 0.9%Pt_1_+Pt_n_/Cu-CuZrO_x_ was pretreated by N_2_O to poison the surface Cu sites (0.9%Pt_1_ + Pt_n_/Cu-CuZrO_x_-N_2_O), sharply-declined glycerol conversion to 70.0% further supporting the enhanced C-H activation by atomic Pt_1_ sites (Supplementary Table 8). To ensure that the catalytic experiments were performed under the regime of kinetic control, Mears and Weisz–Prater analyses^62–65^ were employed to investigate the mass transfer based on the reaction rates in our work (Supplementary Table 9). The calculated values of the Mears criterion are 7.74 × 10^−5^, 5.57 × 10^−5^, and 4.31 × 10^−5^ on 0.9%Pt_1_ + Pt_n_/Cu-CuZrO_x_, 0.9%Pt_n_/Cu-CuZrO_x_, and 0.9%PtCu-CuZrO_x_, respectively, far smaller than 0.15, implying that external mass transfer effects can be neglected. The calculated values of the Weisz–Prater Criterion are 2.96 × 10^−9^, 2.32 × 10^−9^, and 1.18 × 10^−9^ on 0.9%Pt_1_ + Pt_n_/Cu-CuZrO_x_, 0.9%Pt_n_/Cu-CuZrO_x_ and 0.9%PtCu-CuZrO_x_, respectively, far smaller than 1, implying that internal mass transfer effects can also be neglected.

Over 0.9%Pt_1_+Pt_n_/Cu-CuZrO_x_, the GLYA selectivity holds at 80.4% along the increasing time, with a small amount of glyceraldehyde (5.3%) and DHA (9.5%) (Supplementary Fig. 14a). No obvious GLAD accumulation or transformation is observed along with the increasing reaction time, indicating a fast conversion from GLAD to GLYA. GLYA selectivity over 0.9%Pt_n_/Cu-CuZrO_x_ slightly increases along with the reaction time and reaches 66.7% at 4 h with 10.6% GLAD, 11.9% of DHA, and 5.4% of acetic acid (HAc) (Supplementary Fig. 14b). Over 0.9%PtCu-CuZrO_x_, it gradually increases and reaches 60.6% at 6 h with 9.5% of GLAD, 12.6% of DHA, and 9.6% of HAc (Supplementary Fig. 14c). An obvious transformation from GLAD to GLYA is observed along with the increasing time over 0.9%Pt_n_/Cu-CuZrO_x_ and 0.9%PtCu-CuZrO_x_, demonstrating much slower rate of the GLAD oxidation to GLYA than that from glycerol to GLAD. At similar glycerol conversion (Fig. 9c), the GLYA selectivity over 0.9%Pt_n_/Cu-CuZrO_x_ (66.7%) and 0.9%PtCu-CuZrO_x_ (60.6%) is much lower than that over 0.9%Pt_1_ + Pt_n_/Cu-CuZrO_x_ (80.2 ± 0.2%), ascribed to the poorer transformation from GLAD to GLYA and more C-C cleavage to HAc. Over 0.9%Pt_1_ + Pt_n_/Cu-CuZrO_x_-N_2_O, a sharp decline in the GLYA selectivity to 67.9% with an increased GLAD selectivity of 18.2% confirms that Pt^δ-^-Cu bonds play a key role in the C-H activation from GLAD to GLYA. Moreover, it is worth noting that similar DHA selectivity over 0.9%Pt_1_ + Pt_n_/Cu-CuZrO_x_ (10.0 ± 0.5%), 0.9%Pt_n_/Cu-CuZrO_x_ (11.9%), and 0.9%PtCu-CuZrO_x_ (12.6%) was detected, respectively (Supplementary Table 8). According to our recent work^66^, an isotope labeling experiment using deuterium labeling of glycerol in the secondary C-H bond was performed and DHA was side-produced from the direct activation of secondary O-H bonds in glycerol (Supplementary Fig. 15).

The selective oxidation of GLAD to GLYA was then carried out. A GLAD conversion of 98.0% with a GLYA selectivity of 93.1% over 0.9%Pt_1_ + Pt_n_/Cu-CuZrO_x_ at 5 h is achieved, much more efficient than that over 0.9%Pt_n_/Cu-CuZrO_x_ (71.1% of GLAD conversion with 81.2% of GLYA selectivity) and 0.9%PtCu-CuZrO_x_ (65.2% of GLAD conversion with 64.9% of GLYA selectivity), respectively (Fig. 9d, e). It could well account for the steady GLYA selectivity over 0.9%Pt_1_+Pt_n_/Cu-CuZrO_x_ in the cascade oxidation of glycerol to GLYA. In the profile of the reaction rate towards the Pt valence state (Fig. 9f, solid ball), increased with the Pt valence state, the reaction rate sharply increases, implying that the GLAD conversion mainly depends on the electron-rich state of the C-H activation as well as the insertion of O-H bonds. The synergistic activation of GLAD in η^2^-mode and the enhanced C-H activation are reasonably inferred to enhance the GLAD conversion. In addition, the established H_2_O_2_ in the reaction was then quantified using KMnO_4_ titration and then was related to the Pt valence state (Fig. 9f, hollow ball). A similar increase tendency is displayed along with the increasing Pt valence state. It demonstrates that the generated reactive oxygen species during the whole oxidation process were sufficient to accomplish the cascade oxidation. Benefited from the superiority of both the adsorption of aldehyde and the desorption of carboxylic acid in situ FT-IR spectra (Figs. 5–7), the enhanced GLYA selectivity could be reasonably achieved.

The selective oxidation of glycerol over 0.9%Pt_1_ + Pt_n_/Cu-CuZrO_x_ under tailored reaction conditions has been performed (Supplementary Table 10). Under the O_2_ flow rate of 30 (entry 1), 60 (entry 2), or 150 mL/min (entry 3), the glycerol conversion and GLYA selectivity hold at the same value, further confirming the absence of the mass transfer. Under the glycerol/Pt (mol/mol) of 300 (entry 1), 500 (entry 4), 650 (entry 5), and 1000 (entry 6), the glycerol conversion gradually declined at a similar reaction rate between 157–160 mol_gl_ mol_Pt_^−1^ h^−1^ and the GLYA selectivity slightly increases with decreased GLAD and DHA selectivity. Under the glycerol/Pt (mol/mol) of 1000, a GLYA selectivity of 84.4% at a glycerol conversion of 79.8% is obtained. Moreover, under increased glycerol concentration to 0.3 M, a GLYA selectivity of 82.3% at a declined glycerol conversion of 80.0% is obtained with the reaction rate holding at 158 mol_gl_ mol_Pt_^−1^ h^−1^. No obvious change in the reaction intermediates due to catalyst poisoning was detected. Over 0.9%Pt_1_ + Pt_n_/Cu-CuZrO_x_, the obtained glycerol conversion and GLYA selectivity in the selective oxidation of glycerol to GLYA at 60 ^o^C under atmospheric pressure in an O_2_ flow, to the best of our knowledge, is higher than the reports under the same condition (Supplementary Table 11).

The reusability of 0.9%Pt_1_ + Pt_n_/Cu-CuZrO_x_ was then tested under the glycerol/Pt (mol/mol) of 1000 (Supplementary Fig. 16). The catalyst was separated by simple filtration and subsequently used without any treatment. Both the glycerol conversion and the GLYA selectivity are well preserved after five runs (Supplementary Fig. 16a). Almost no Pt leaching from the solid catalyst or no Pt presence in the spent reaction solution was detected (Supplementary Fig. 16b). 4.7 and 3.8% of Cu and Zr were detected to leach from the solid in five runs. The total Cu and Zr leached into the collected spent reaction solution for five runs were determined to be 4.2 and 3.6%, consistent with the loss in a solid catalyst. The HRTEM images, the AC-HAADF-STEM images, and the Pt L3-edge FT-EXAFS spectra of the spent 0.9%Pt_1_ + Pt_n_/Cu-CuZrO_x_ (Supplementary Figs. 17 and 18) indicate that the dispersion, the coordination and the electronic state of Pt active sites have been well retained compared to the fresh catalyst. In the Cu 2*p*3/2 XPS spectra and X-ray induced Cu Auger electron spectra of the spent Pt_1_ + Pt_n_/Cu-CuZrO_x_ (Supplementary Fig. 19), the electronic state of Cu species displays no obvious change. The surface Pt/Cu molar ratio increases from 1/15 in the fresh 0.9%Pt_1_ + Pt_n_/Cu-CuZrO_x_ to 1/14 and the Cu^II^/Cu^0/I^ molar ratio decreases from 1/4 to 1/5. Considering no Pt leaching is detected, the slight Cu leaching is deduced to be originated from the Cu^II^ species by the chelation of acid production.

In conclusion, this work proposes and confirms a cascade synergistic catalysis of atomic Pt_1_ and cluster Pt_n_ for the cascade oxidation of glycerol to GLYA, employing Pt_1_ + Pt_n_/Cu-CuZrO_x_ integrated by atomic Pt_1_ and cluster Pt_n_. Compared to the cluster/nanoparticles Pt sites, the enhanced C-H activation in the cascade oxidation by the atomic Pt_1_ in the synergistic catalysis contributes to the simultaneous high glycerol activity and high GLYA selectivity. This work not only paves an alternative strategy for promoting the cascade catalysis by engineering the surface metal active sites but also presents a green and efficient value-added routine from glycerol as industrial by-products. Further work in our lab is still underway to explore multi-synergistic catalysis to further realize the accurate activation in the primary position of polyols.

## Methods

### General information

Unless otherwise noted, all chemicals were purchased and used without further purification.

### Catalyst preparation

As a precursor, the mixed copper and zirconium oxide was prepared by a hydrothermal method^67^. Typically, a mixture solution of 150 mL of Cu(NO_3_)_2_·6H_2_O (0.03 mol) and ZrOCl_2_·8H_2_O (0.015 mol) aqueous solution, and 150 mL of CO(NH_2_)_2_ (0.113 mol) aqueous solution were stirred in a three-necked flask for 0.5 h, and then sealed in a Teflon-lined stainless-steel autoclave, followed by aging at 200 ^o^C for 48 h. The resulting precipitate was centrifuged, washed with deionized water, and dried at 60 ^o^C overnight. The solid was calcined in air at 450 ^o^C (heating rate: 5 ^o^C min^−1^) for 2 h and then cooled down to produce Cu_1_Zr_4.3_O_9.6_. Supported Cu sample was prepared by a reduction treatment of Cu_1_Zr_4.3_O_9.6_ in an H_2_ stream at 350 ^o^C (heating rate: 5 ^o^C min^−1^) for 2 h and then cooled down to room temperature to produce Cu-CuZrO_x_. Supported PtCu samples with varied Pt loadings were prepared via a galvanic replacement method, as illustrated in Supplementary Experimental details. In a typical procedure, the fresh Cu-CuZrO_x_ sample (0.5 g) was dispersed in deoxygenated deionized water (5 mL), followed by dropwise adding desired concentration of H_2_PtCl_6_ solution (1 mL) in the N_2_ atmosphere under vigorous stirring (700 rpm) and then under reflux for 2 h. The resulting slurry was centrifuged, washed thoroughly with deoxygenated deionized water and dried in a vacuum at 60 ^o^C, followed by a reduction in an H_2_ stream at 450 ^o^C (heating rate: 5 ^o^C min^−1^) for 2 h, producing supported PtCu samples. By modulating the concentration of H_2_PtCl_6_ solution in a range from 0.05, 0.2, 0.4, and 1.0 mol/L, supported PtCu samples with Pt loading of 0.5 and 0.9 wt% were obtained, named as 0.5%Pt_1_/Cu-CuZrO_x_ and 0.9%Pt_1_ + Pt_n_/Cu-CuZrO_x_, respectively, combined with the structural identification.

As reference samples, 0.9%Pt_n_/Cu-CuZrO_x_ was prepared by incipient wetness impregnation to introduce PtCl_6_^2-^ onto the Cu surface of Cu-CuZrO_x_ at a pH of 7.0 with a Pt loading of 0.9 wt% followed by a reduction. Considering the isoelectric point of ZrO_2_ (pI = 6.0^68^), an electrostatic repulsion with support makes PtCl_6_^2−^ anions tend to adsorb on Cu nanoparticles. 0.9%Pt_n_/Cu-CuZrO_x_ and 0.9%PtCu-CuZrO_x_ were prepared by an incipient wetness method through impregnating PtCl_6_^2−^ with identical Pt loading of 0.9%Pt_1_ + Pt_n_/Cu-CuZrO_x_ on Cu-CuZrO_x_ and Cu_1_Zr_4.3_O_9.6_, respectively, followed by the same reduction procedure with 0.9%Pt_1_ + Pt_n_/Cu-CuZrO_x_. As control, 0.9%Pt_1_ + Pt_n_/Cu-CuZrO_x_ was treated in a flow of N_2_O/Ar (v:v = 1:9) for 1 h to produce 0.9%Pt_1_ + Pt_n_/Cu-CuZrO_x_-N_2_O.

### Characterizations

XRD measurements were performed on a Shimadzu XRD-6000 diffractometer using Cu Kα radiation (λ = 0.1541 nm) and operated at 40 kV and 30 mA. XRD patterns were collected with a scanning angle (2θ) range of 3°−80° at a scan speed of 5° min^−1^. Quantitative analysis for metal elements was performed on a Shimadzu ICPS-7500 inductively coupled plasma emission spectrometer (ICP-ES). Typically, approximately 2.0 mg of solid was dissolved in aqua regia and diluted to 10 mL in a volumetric flask before characterization. N_2_ adsorption was performed on a Quantachrome Autosorb-1C-VP analyzer to determine the specific surface area and the porosity. The specific surface area was calculated by the BET method. The solid was outgassed in a flow of N_2_ at 100 °C for 8 h prior to the measurement. H_2_-TPR was carried out on a Micrometric ChemiSorb 2750 chemisorption instrument with a TCD detector. FESEM image was collected on a ZEISS Supra-55 scanning electron microscope. TEM and HRTEM images were collected on a Tecnai G2 F30 S-TWIN transmission electron microscope operated at 300 kV. AC-HAADF-STEM) and EDS images were taken on a JEM-ARM 200F electron microscope capable of sub-angstrom resolution. XPS characterization was performed on a Shimadzu KRATOS AXIS SUPRA at a pressure of 2 × 10^−9^ Torr. The C1*s* peak at 284.6 eV was used for calibration. EXAFS measurement at the Pt K-edge was performed at the beamline 1W1B of the Beijing Synchrotron Radiation Facility (BSRF), Institute of High Energy Physics (IHEP), and the Chinese Academy of Sciences (CAS). The catalyst particle size distribution was measured on a Mastersizer 2000 laser particle size analyzer.

### In situ FT-IR study

The adsorption/desorption of 1-propanol, acetaldehyde, and propionic acid on supported PtCu samples were recorded on a Nicolet iS50 spectrometer equipped with a cell fitted with BaF_2_ windows and an MCT-A detector cooled by liquid nitrogen. The spectrum was collected at a resolution of 4 cm^−1^ with an accumulation of 64 scans in the range of 4000–750 cm^−1^. Approximately 20 mg of solid sample was pressed into a self-supported wafer in each measurement. Then, the sample wafer was pretreated under a flow of H_2_/Ar (v:v = 1:4) for 30 min at 300 °C to remove the impurities absorbed on the surface and then cooled to 20 °C. The background was collected at 20 °C under a flow of Ar (40 mL min^−1^) before the adsorbate introduction. 1-propanol, acetaldehyde, or propionic acid was introduced by bubbling pristine adsorbate liquid by a flow of Ar (40 mL min^−1^) at 20 °C for 30 min to obtain a stable spectrum. Then, the system was purged with a flow of Ar (40 mL min^−1^), and the desorption spectra were recorded towards the desorption time until there was no change in the band intensity. For in situ time-resolved FT-IR spectra of 0.9%Pt_1_ + Pt_n_/Cu-CuZrO_x_ on exposure to 1-propanol at 60 ^o^C in a flow of O_2_ and H_2_O. Adsorbates were introduced to the chamber by bubbling 1-propanol aqueous solution (water content: 1 wt%) by a flow of O_2_/Ar (v:v = 1:5). As for the adsorption of CO, the sample was exposed to a flow of CO/He (v:v = 1:99) at 20 ^o^C for 30 min. The adsorption of glycerol and glyceraldehyde on supported PtCu samples were recorded on a VERTEX70V FT-IR spectrometer equipped with a cell fitted with BaF_2_ windows and a photovoltaic MCT detector. Considering the high boiling point, glycerol or glyceraldehyde was pre-heated at 60 ^o^C and then introduced by a flow of Ar (40 mL min^−1^) for 5 min. The spectra were collected at 25 ^o^C. To avoid the adsorbate condensation, the BaF_2_ windows should be pretreated at 70 ^o^C.

### Catalytic evaluation

The catalytic oxidation of glycerol/GLAD was conducted in an ebullated bed. In a typical procedure, 15 mL of an aqueous glycerol solution (0.1 M) and a proper amount of catalyst in powder form with an initial molar glycerol/Pt ratio of 300 were loaded into an ebullated bed. During the oxidation reaction, O_2_ (99.9%) was introduced into the reactor at 30 mL min^−1^ via a mass flow controller at atmospheric pressure. The reaction time of zero was defined when the temperature of the reaction mixture reached 60 ^o^C (heating rate: 10 ^o^C min^−1^). After the reaction, the liquid products were analyzed by an Agilent LC-1260 HPLC equipped with both UV detector and RID detector was used^69^. An Aminex HPX-87 H column (Bio-Rad, 300 × 7.8 mm) operating at 50 °C was used with 10 mM aqueous H_2_SO_4_ or HCOOH as the eluent at a flow of 0.5 mL min^−1^. For quantification, an external calibration method was used. The gaseous product was collected and identified on a Shimadzu 2014C GC equipped with a TDX-1 column and TCD detector. CO_2_ was identified as the only gaseous product. Catalytic performance towards the selective oxidation of glycerol over 0.9%Pt_1_ + Pt_n_/Cu-CuZrO_x_ under tailored reaction conditions was carried out. 0.9%Pt_1_ + Pt_n_/Cu-CuZrO_x_ was recycled by simple filtration and subsequently used in the next catalytic cycle without further purification or any treatment. The catalytic oxidation of GLAD was conducted in a similar procedure.

### Labeling experiments

Reactions with deuterium-labeled glycerol, ^18^O_2_ gas, and H_2_^18^O were performed using 3 mL of glycerol and 0.01 g of catalyst. A liquid chromatograph equipped with a Quattro Premier XE mass spectrometer was used to identify the formed labeled products. To verify the formation of H_2_O_2_, a mixed solution (abbreviated as P) of phosphate buffer, *N*, *N*-diethylbenzene-1,4-diamine sulfate, and horseradish peroxidase was added to a fresh reaction solution after catalyst separation. Reference solutions of H_2_O_2_ (0.05 M), glycerol (0.1 M), GLYA (0.1 M), GLAD (0.1 M), DHA (0.1 M), TTA (0.1 M), GCA (0.1 M), OA (0.1 M), HAc (0.1 M), and FA (0.1 M) were used.

## Supplementary information

Supplementary Information

## Data Availability

The data supporting this study are available within the paper and the Supplementary Information. All other relevant source data are available from the corresponding author upon reasonable request.
