# Supplementary material for: Pt1 enhanced C-H activation synergistic with Ptn catalysis for glycerol cascade oxidation to glyceric acid
Source: Nat Commun. 2022 Sep 17;13:5467. doi: 10.1038/s41467-022-33038-w (PMC9482651; doi:10.1038/s41467-022-33038-w)
Supplement: Supplementary file 1 — Supplementary Information [file 41467_2022_33038_MOESM1_ESM.pdf]

Supplementary Information

Pt_1 Enhanced C-H Activation Synergistic with Pt_n Catalysis for Glycerol Cascade Oxidation to Glyceric Acid

Zhe An,[1] Zilong Zhang,[1] Zeyu Huang,[1] Hongbo Han,[1] Binbin Song,[1] Jian Zhang,[1] Qi Ping,[1] Yanru Zhu,[1] Hongyan Song,[1] Bin Wang,[2] Lirong Zheng,[3] and Jing He*[1]

[1] *State Key Laboratory of Chemical Resource Engineering, Beijing University of Chemical Technology, Beijing 100029, P. R. China.*

[2] *Beijing Research Institute of Chemical Industry, Sinopec Group, 100013 Beijing, P. R. China.*

[3] *Technology Institute of High Energy Physics, Chinese Academy of Sciences, Beijing 100049, P. R. China.*

Corresponding Author

**e-mail: jinghe@263.net.cn.*

Table of contents

I. Supplementary Figures 4

Supplementary Fig. 1 4

Supplementary Fig. 2 5

Supplementary Fig. 3 6

Supplementary Fig. 4 7

Supplementary Fig. 5 8

Supplementary Fig. 6 9

Supplementary Fig. 7 10

Supplementary Fig.8 11

Supplementary Fig. 9 12

Supplementary Fig. 10 13

Supplementary Fig. 11 14

Supplementary Fig. 12 15

Supplementary Fig. 13 16

Supplementary Fig. 14 17

Supplementary Fig. 15 18

Supplementary Fig. 16 19

Supplementary Fig. 17 20

Supplementary Fig. 18 21

Supplementary Fig. 19 22

II. Supplementary Tables 23

Supplementary Table 1 23

Supplementary Table 2 24

Supplementary Table 3 25

Supplementary Table 4 26

Supplementary Table 5 27

Supplementary Table 6 28

Supplementary Table 7 29
Supplementary Table 8 30
Supplementary Table 9 31
Supplementary Table 10 32
Supplementary Table 11 33
III. Supplementary Experimental details 34
1. The details of galvanic replacement method 34
2. Analysis method for the reaction liquid 34
3. Analysis method for the gaseous product 37
IV. Supplementary References 39

I. Supplementary Figures

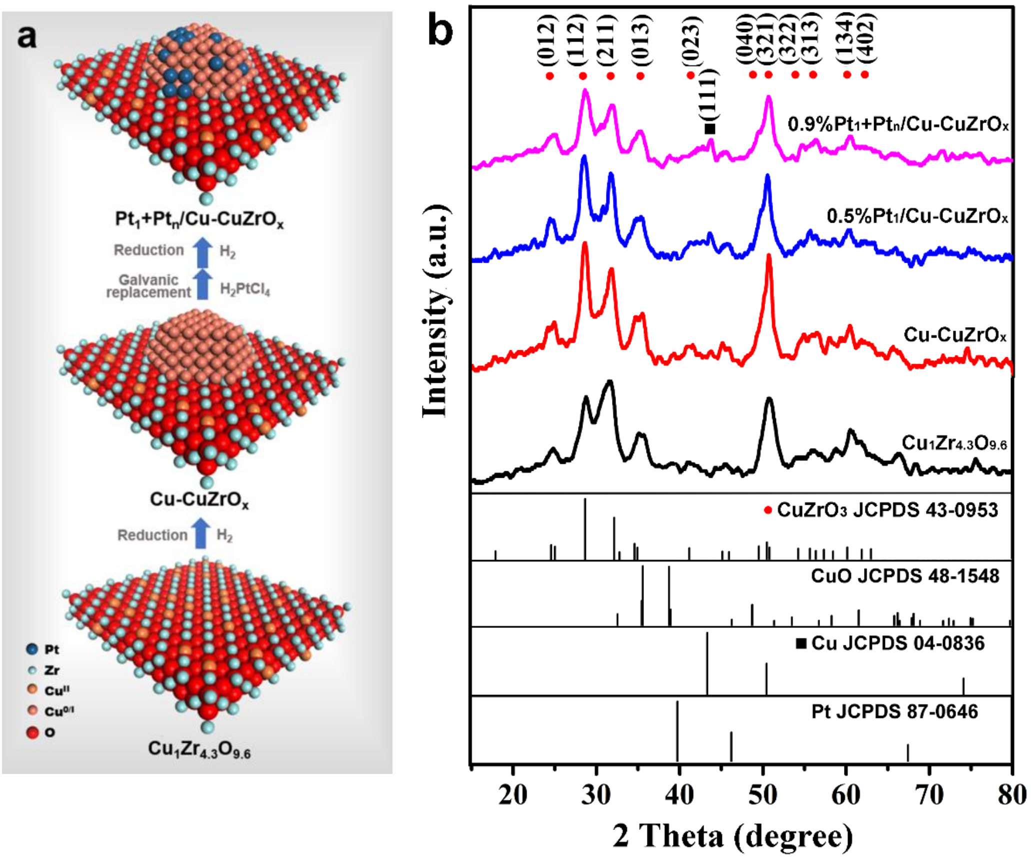

Supplementary Fig. 1 Preparation and crystalline structure of catalysts. a Schematic illustration for the preparation of Pt_1+Pt_n/Cu-CuZrO_x. **b** XRD patterns of precursors and catalysts.

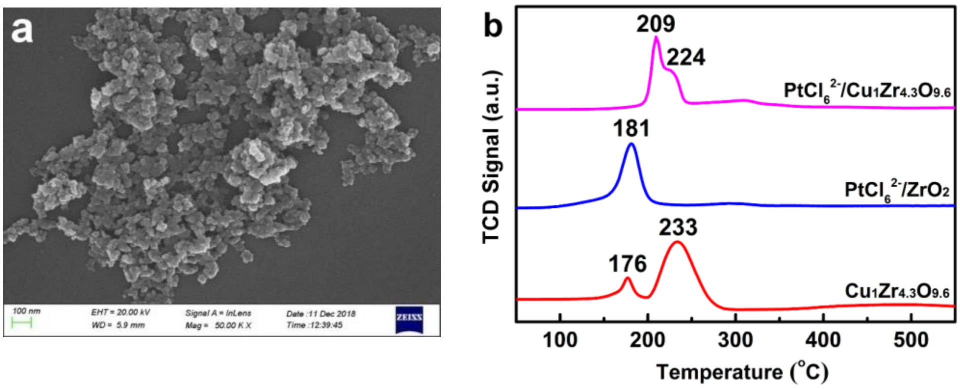

Supplementary Fig. 2 Morphology and H_2-TPR of precursors. a FESEM image of $Cu_1Zr_{4.3}O_{9.6}$. **b** H_2-TPR profiles of $Cu_1Zr_{4.3}O_{9.6}$, $PtCl_6^{2-}/ZrO_2$, and $PtCl_6^{2-}/Cu_1Zr_{4.3}O_{9.6}$.

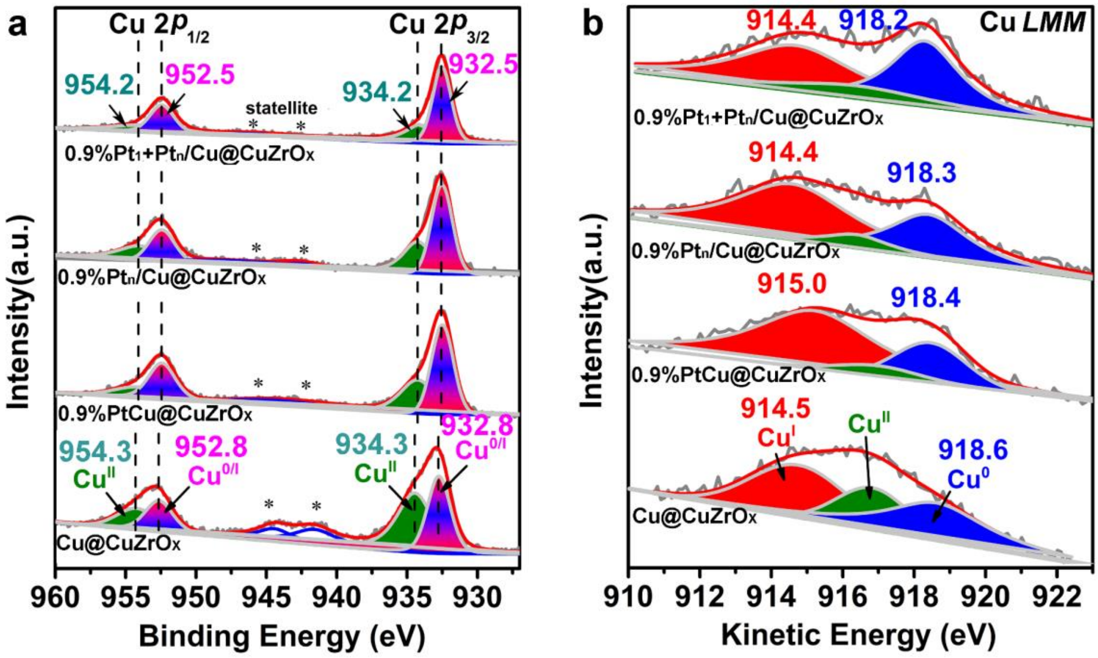

Supplementary Fig. 3 Electronic structure analysis from XPS characterization. Cu 2*p* core-level XPS spectra **(a)** and X-ray induced Cu Auger electron spectra **(b)** of 0.9%Pt_1+Pt_n/Cu-CuZrO_x, 0.9%Pt_n/Cu-CuZrO_x, 0.9%PtCu-CuZrO_x, and Cu-CuZrO_x.

Cu XPS 2*p*3/2 spectra (**Supplementary Fig. 3a**) for Cu-CuZrO_x could be deconvoluted into two peaks with the binding energy for one at 932.8 eV assigned[1] to Cu^0 or Cu^I species, and another at 934.3 eV to Cu^{II} species[1]. The presence of Cu^{II} species is ascribed to the un-reduced Cu^{II} in CuZrO_x, which coincides with the HADDF-STEM EDS element mapping (**Fig. 2c and 2f**). After Pt loading, the peak for Cu^0 or Cu^I species shifts to lower binding energy of 932.5 eV, indicating the electron donation of Cu to Pt. X-ray induced Cu Auger electron spectra (**Supplementary Fig. 3b**) was employed to further clarify the Cu^0 and Cu^I species. Besides the Cu^{II} Auger L3M45M45 excited mode located at around 917.0 eV (**filled in olive**), the fitted peaks located at 914.5~915.0 eV are attributed to the Cu^I Auger L3M45M45 excited mode (**filled in red**) and the peaks at 918.2~918.6 eV are attributed to the Cu^0 Auger L3M45M45 excited mode (**filled in blue**) according to the previous reports[2]. The kinetic energy (915.0 eV) of Cu^I species in 0.9%PtCu-CuZrO_x is higher than that in 0.9%Pt_1+Pt_n/Cu-CuZrO_x (914.4 eV), 0.9%Pt_n/Cu-CuZrO_x (914.4 eV), and Cu-CuZrO_x (914.5 eV). It is deduced that the presence of Cu^I species is ascribed to Cu species located at the interfaces between supported PtCu or Cu nanoparticles and CuZrO_x. For Cu^0 species, compared with Cu-CuZrO_x (918.6 eV), the kinetic energy shifts to lower energy for 0.9%PtCu-CuZrO_x (918.4 eV), 0.9%Pt_n/Cu-CuZrO_x (918.3 eV), and 0.9%Pt_1+Pt_n/Cu-CuZrO_x (918.2 eV), respectively, confirming the electron-deficient state for Cu sites in the Pt-Cu bonds of the three catalysts.

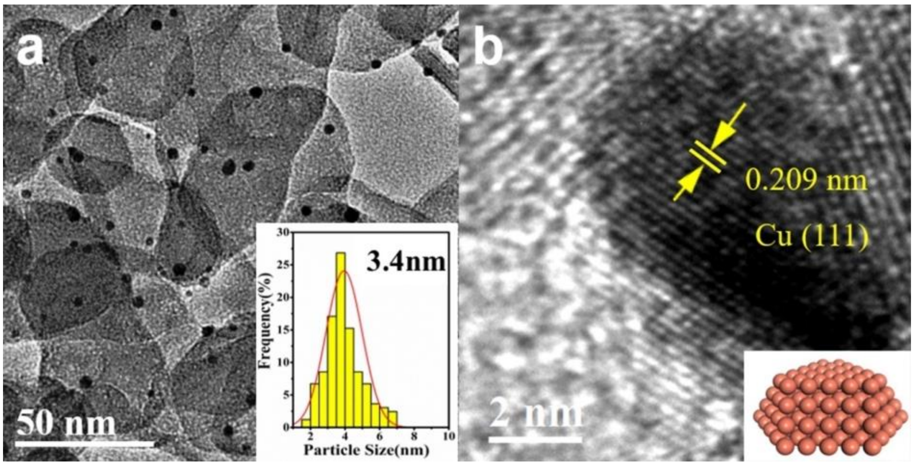

Supplementary Fig. 4 Structural characterization of Cu-CuZrO$_x$. Representative HRTEM images and the corresponding particle size distribution of Cu nanoparticles. Scale bars: **a** 50 nm; **b** 2 nm.

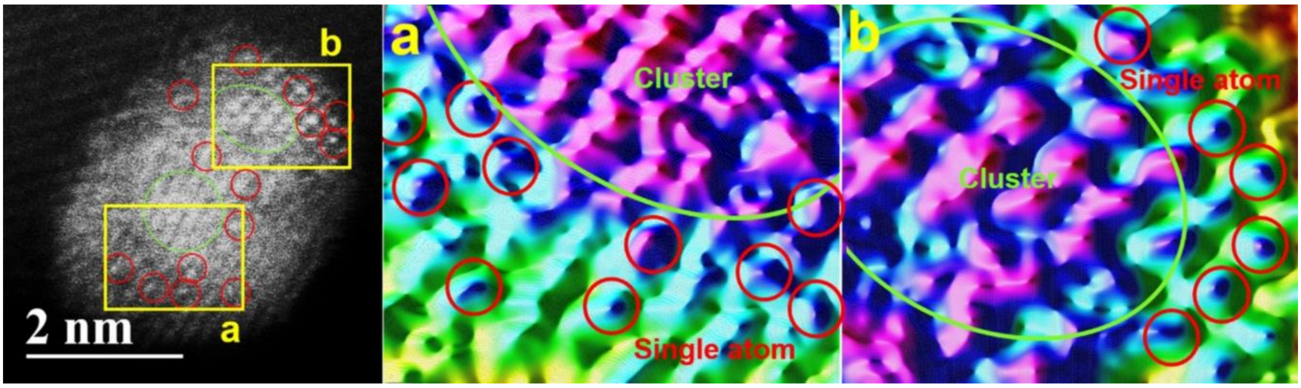

Supplementary Fig. 5 Coexistence of adjacent Pt_1 and Pt_n sites. Representative AC-HAADF-STEM images of 0.9%Pt_1+Pt_n/Cu-$CuZrO_x$ and the corresponding 3D surface simulation of the marked regions in yellow rectangles **(a,b)** by the software Fiji.

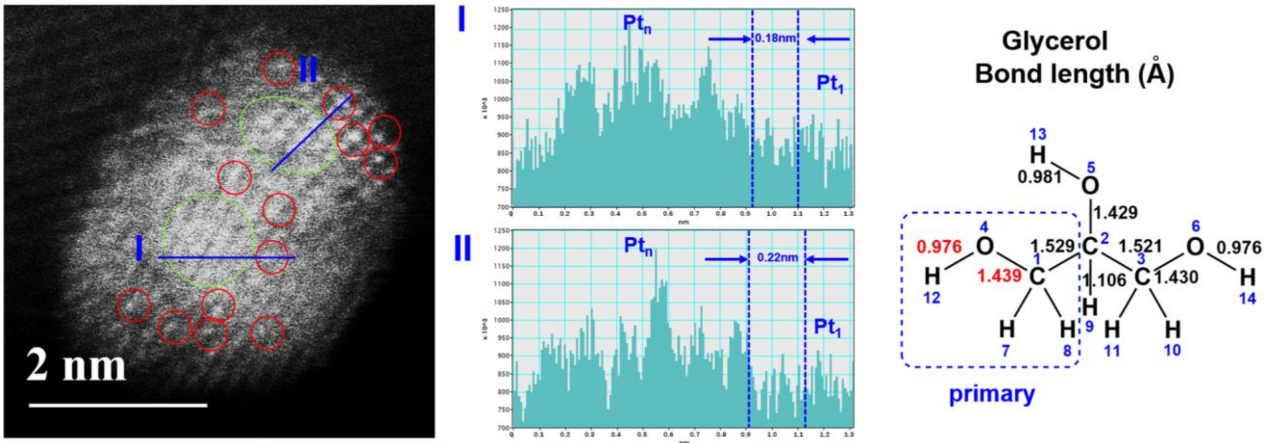

Supplementary Fig. 6 Identification of adjacent Pt_1 and Pt_n sites. Representative AC-HAADF-STEM images of 0.9%Pt_1+Pt_n/Cu-$CuZrO_x$ and the corresponding brightness intensity profiles **(line I and line II)**. The bond length of glycerol is displayed according to the Ref. 3.

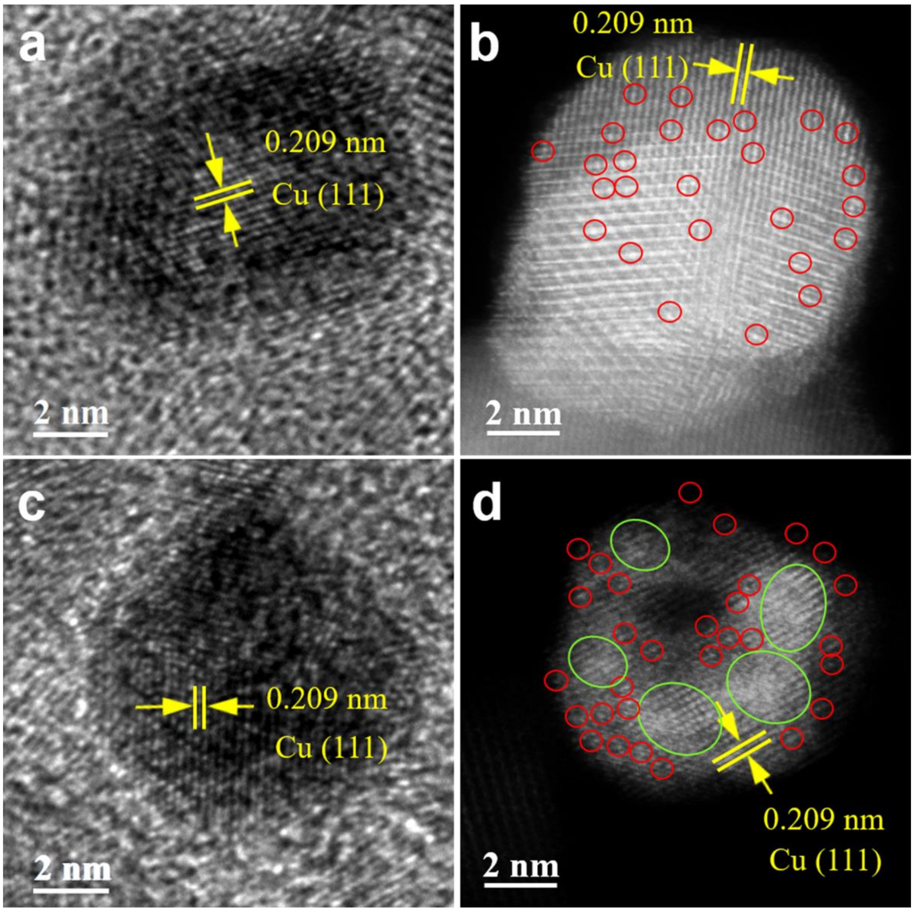

Supplementary Fig. 7 Structural characterization of 0.5%Pt_1/Cu-$CuZrO_x$ and 0.9%Pt_1+Pt_n/Cu-$CuZrO_x$. Representative HRTEM (**a,c**) and AC-HAADF-STEM (**b,d**) images of 0.5%Pt_1/Cu-$CuZrO_x$ (**a,b**) and 0.9%Pt_1+Pt_n/Cu-$CuZrO_x$ (**c,d**).

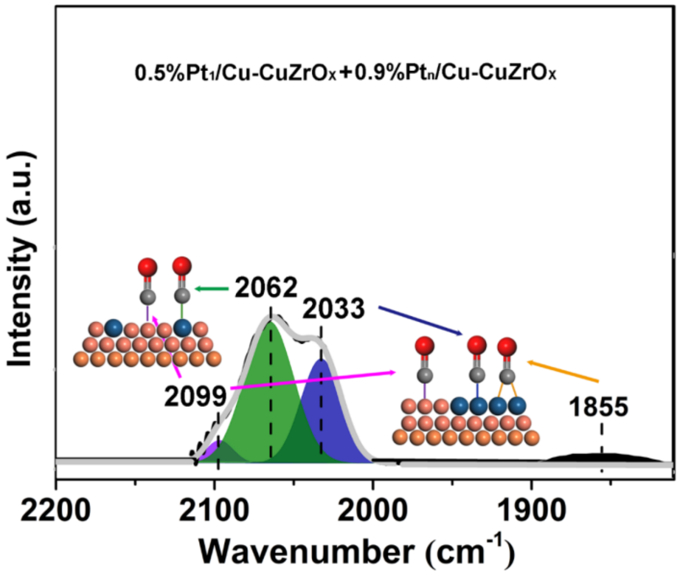

Supplementary Fig.8 CO-DRIFTS spectra of the physically-mixed 0.5%Pt_1/Cu-$CuZrO_x$ and 0.9% Pt_n/Cu-$CuZrO_x$. Besides the band for CO adsorbed on Cu^0 species (2099 cm^{-1}), the bands for CO linearly-adsorbed on atomic Pt in 0.5%Pt_1/Cu-$CuZrO_x$ (2062 cm^{-1}), and CO linearly- and bridged-adsorbed on Pt clusters in 0.9%Pt_n/Cu-$CuZrO_x$ (2033 and 1855 cm^{-1}) are observed.

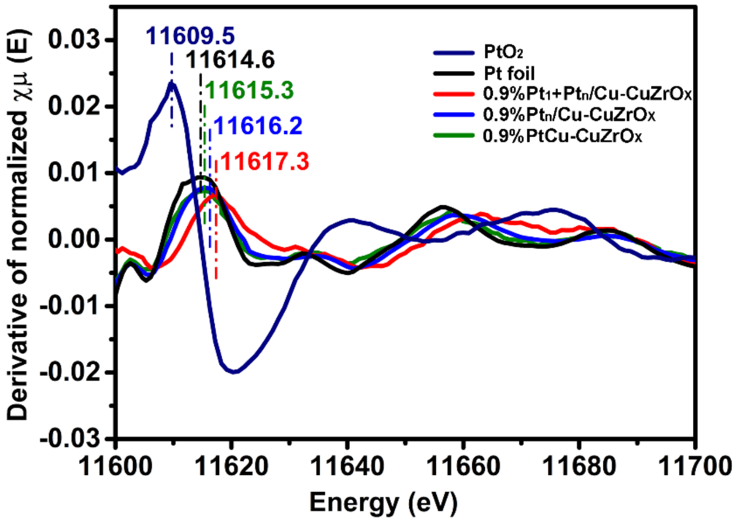

Supplementary Fig. 9 Pt electronic structure analysis from XANES spectra. First-derivative Pt L3-edge XANES spectra of 0.9%Pt_1+Pt_n/Cu-$CuZrO_x$, 0.9%Pt_n/Cu-$CuZrO_x$, and 0.9%PtCu-$CuZrO_x$ with bulk Pt foil and PtO_2 as references.

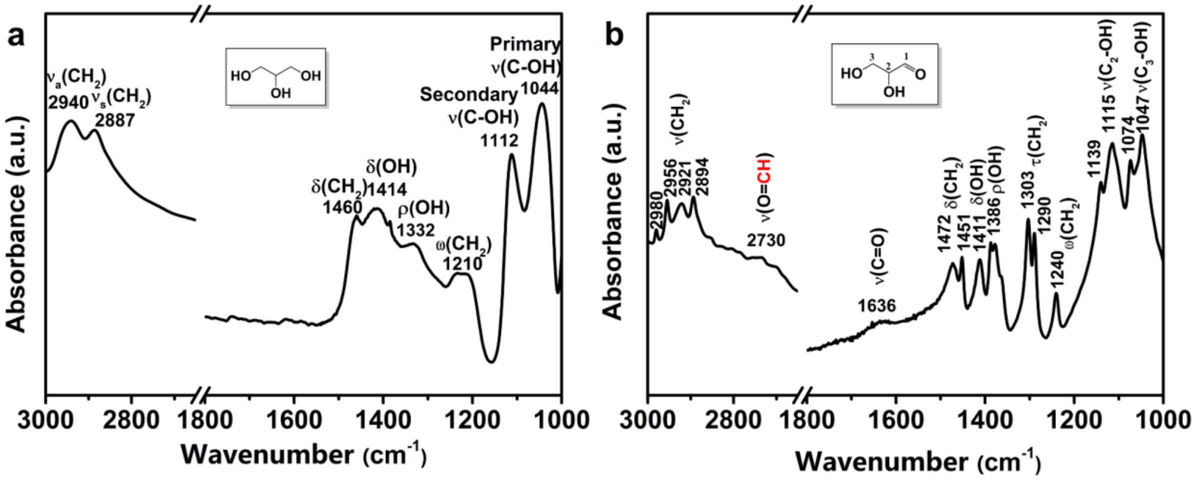

Supplementary Fig. 10 FT-IR spectra of adsorbates for comparison. FT-IR spectra of pristine glycerol **(a)** and glyceraldehyde **(b)** with the corresponding the band assignments.

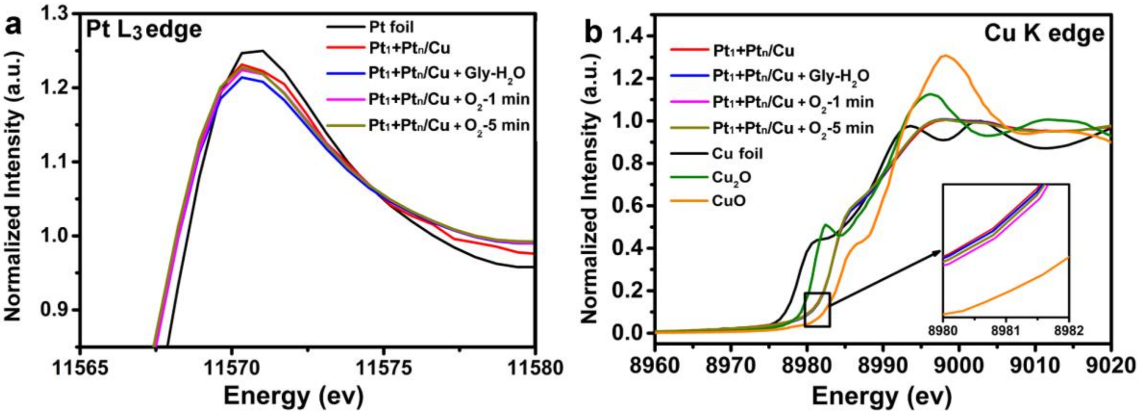

Supplementary Fig. 11 In situ normalized XANES spectra of 0.9%Pt_1+Pt_n/Cu-$CuZrO_x$. Pt L3 edge (**a**) and Cu K edge (**b**) normalized XANES spectra followed by the sequential exposure to glycerol solution and O_2 flow at 1 and 5 min with the reference to bulk Pt foil, Cu foil, Cu_2O, and CuO, respectively.

In in situ Pt L3 edge XANES spectra over 0.9%Pt_1+Pt_n/Cu-$CuZrO_x$ (**Supplementary Fig. 11a**), once the vapor of the glycerol aqueous solution was introduced, the white line peak displayed a distinct shift to low energy compared with the fresh catalyst. When switching to O_2 in 1 and 5 min, Pt species gradually return to higher valence state close to that for the fresh catalyst. As for the Cu K edge XANES spectra (**Supplementary Fig. 11b**), the fresh 0.9%Pt_1+Pt_n/Cu-$CuZrO_x$ shows an absorption edge between Cu_2O and CuO, indicating Cu^+ is the main chemical state with some Cu^0 or Cu^{2+} species, in line with the XPS results (**Supplementary Fig. 3**). Once the vapor of the glycerol aqueous solution was introduced, the Cu adsorption edge shows no significant shift compared with the fresh 0.9%Pt_1+Pt_n/Cu-$CuZrO_x$, demonstrating that the Cu species has not been involved in the glycerol activation. Then O_2 stream was introduced. The adsorption edge of Cu displays a slight shift to higher energy in 1 min, and then returns to low energy in 5 min, which is deduced to be attributed to a small amount of O_2 adsorption on Cu^0 or Cu^I species. When O_2 was exhausted, the Cu electronic state was recovered.

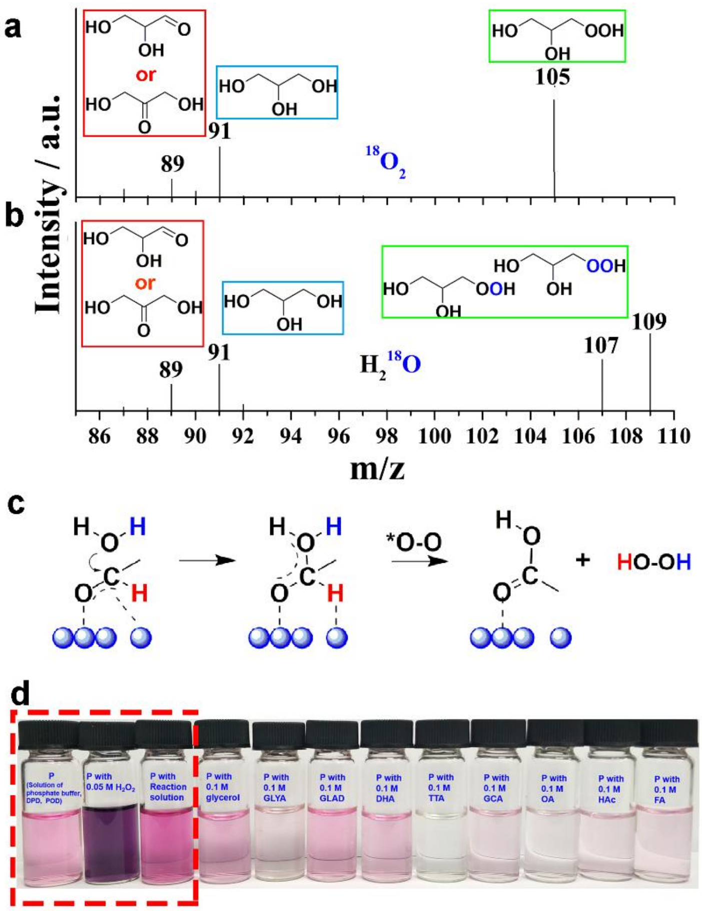

Supplementary Fig. 12 Oxidation mechanism study based on the isotope labelling experiments. **a-c** Mass spectra (electronegative ion mode) of the reaction solution over 0.9%Pt_1+Pt_n/Cu-$CuZrO_x$ in the oxidation of glycerol with $^{18}O_2$ (**a**) and $H_2{}^{18}O$ (**b**) labeling, and the proposed OH insertion process (**c**). Reaction conditions: ebullated bed, 15 mL of substrate aqueous solution (0.1 mol·L^{-1}), substrate/Pt (mol/mol) = 300, 60 ºC, O_2 flow of 30 mL/min. **d** Photographs of a fresh reaction solution and the referential samples mixed with a solution of P containing phosphate buffer, N,N-diethylbenzene-1,4-diamine sulfate (DPD), and horseradish peroxidase (POD).

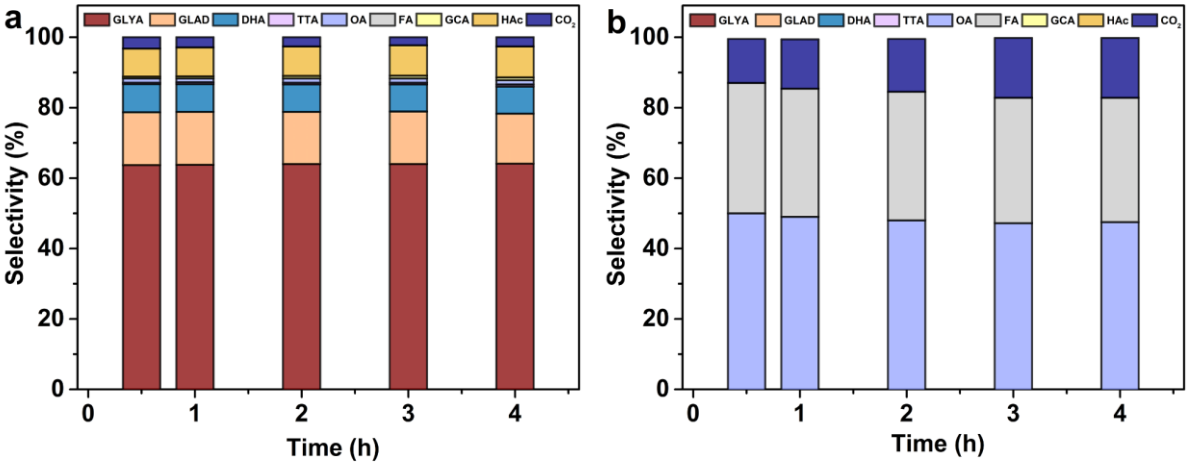

Supplementary Fig. 13 Catalytic performance of 0.5%Pt_1/Cu-CuZrO_x and Cu-CuZrO_x. Time-dependent product selectivity over 0.5%Pt_1/Cu-CuZrO_x (**a**) and Cu-CuZrO_x (**b**). Reaction conditions: ebullated bed, 15 mL of glycerol aqueous solution (0.1 mol·L^{-1}), glycerol/Pt (mol/mol) = 300, 60 ºC, O_2 30 mL/min. Cu-CuZrOx was input using the same mass with 0.9%Pt_1+Pt_n/Cu-CuZrO_x. Abbreviations: GLYA (glyceric acid), GLAD (glyceraldehyde), DHA (dihydroxyacetone), TTA (tartronic), OA (oxalic acid), FA (formic acid), GCA (glycolic acid), and HAc (acetic acid). CO_2 was identified as the only gaseous product.

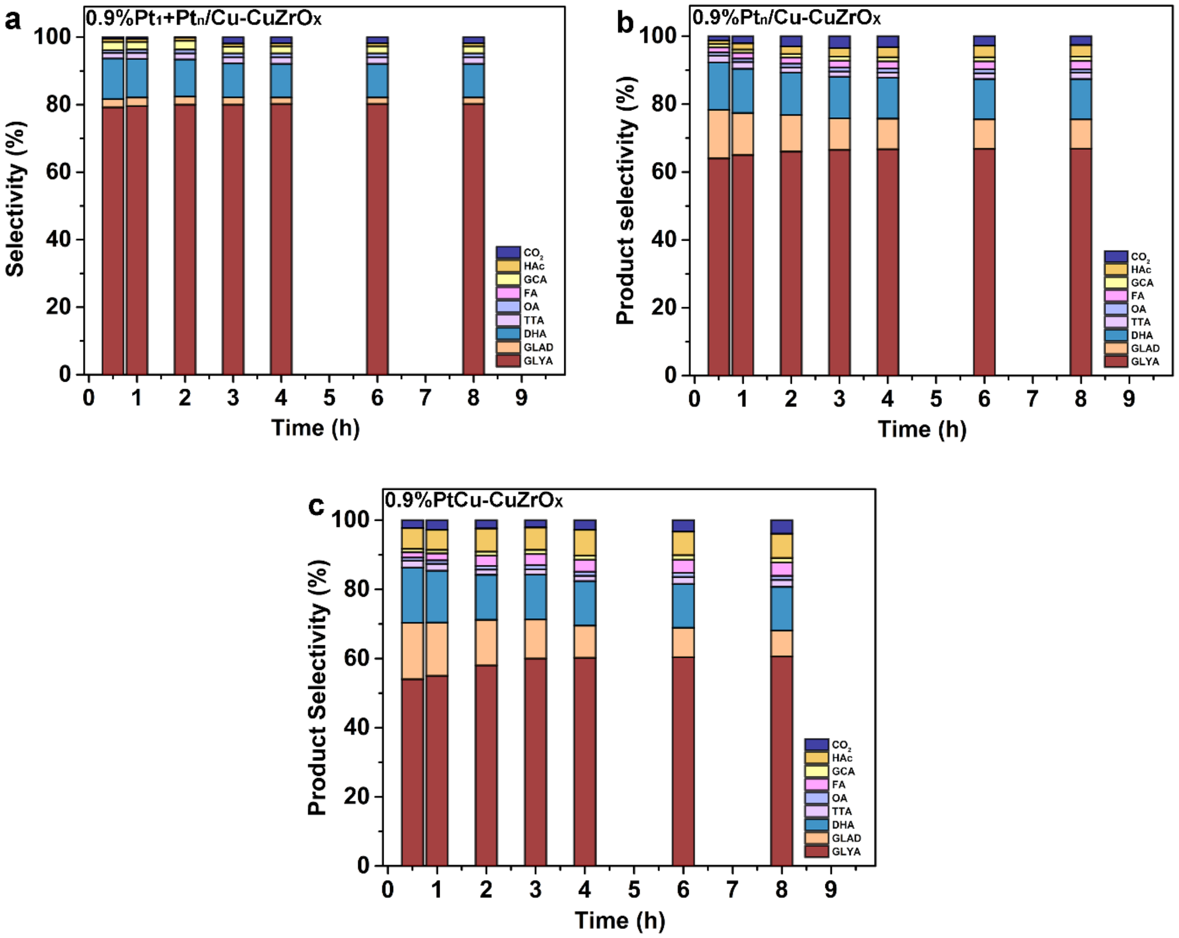

Supplementary Fig. 14 Catalytic performance of PtCu catalysts. Time-dependent product selectivity over 0.9%Pt_1+Pt_n/Cu-CuZrO_x **(a)**, 0.9%Pt_n/Cu-CuZrO_x **(b)**, and 0.9%PtCu-CuZrO_x **(c)**. Reaction conditions: ebullated bed, 15 mL of glycerol aqueous solution (0.1 mol·L^{-1}), glycerol/Pt (mol/mol) = 300, 60 °C, O_2 30 mL/min. Cu-CuZrO_x was input using the same mass with 0.9%Pt_1+Pt_n/Cu-CuZrO_x. Abbreviations: GLYA (glyceric acid), GLAD (glyceraldehyde), DHA (dihydroxyacetone), TTA (tartronic), OA (oxalic acid), FA (formic acid), GCA (glycolic acid), and HAc (acetic acid).

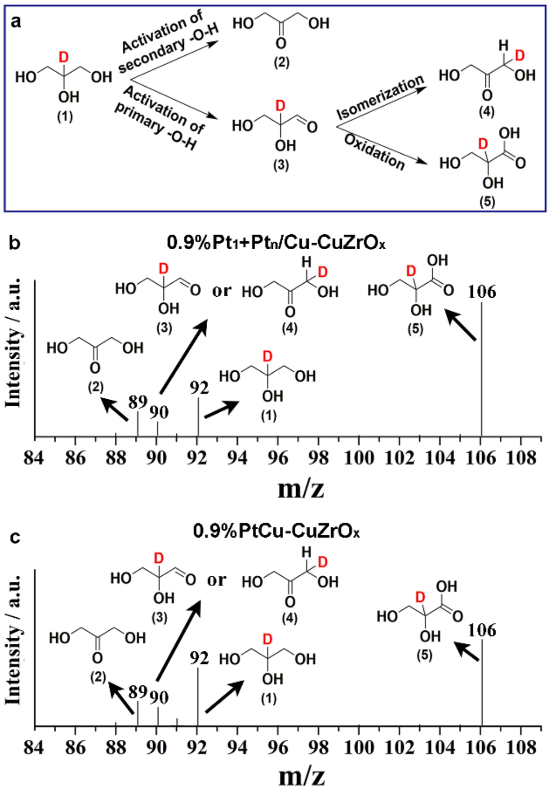

Supplementary Fig. 15 The main intermediates and products initiated by deuterium labeling of glycerol at the secondary C-H bond. Possible reaction pathways (**a**) and the mass spectra of the fresh reaction solution in the oxidation of deuterium-labeled glycerol at the secondary C-H bond over 0.9%Pt_1+Pt_n/Cu-$CuZrO_x$ (**b**) and 0.9%PtCu-$CuZrO_x$ (**c**).

One pathway is the direct activation of secondary O-H and C-D bonds of the labelled glycerol in the secondary C-H bond **(1)**, leading to DHA without deuterium atoms **(2)** (**Supplementary Fig. 15a**). Another pathway is the activation of primary O-H bonds to form a deuterium-labelled GLAD intermediate in the secondary position **(3)**, followed by further oxidation, leading to deuterium-labelled GLYA with C-D bonds in the secondary position **(5)**, or deuterium transfer, leading to deuterium-labelled DHA with C-D bonds in the primary position **(4)** (**Supplementary Fig. 15a**). Over both 0.9%Pt_1+Pt_n/Cu-$CuZrO_x$ (**Supplementary Fig. 15b**) and 0.9%PtCu-$CuZrO_x$ (**Supplementary Fig. 15c**), unlabelled DHA were observed, indicating that DHA more likely originates from the direct activation of secondary O-H and C-H bonds than the isomerization of GLAD according to the relative intensity in the mass spectra.

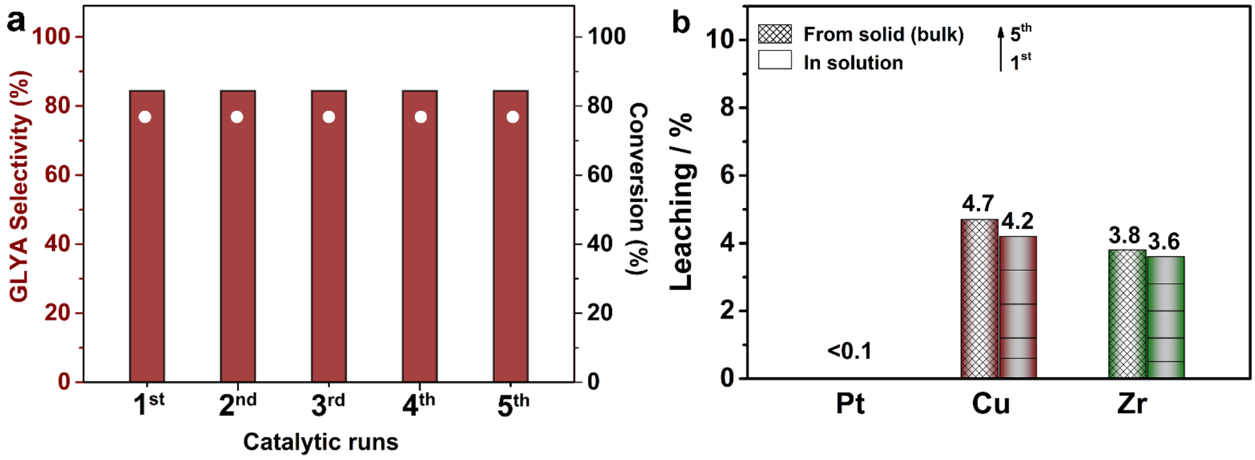

Supplementary Fig. 16 Catalyst reusability and metal leaching. Reusability of 0.9%Pt_1+Pt_n/Cu-CuZrO_x in the selective oxidation of glycerol **(a)** and the total leaching of Pt, Cu, and Zr from solids and in solution in five catalytic runs from ICP **(b)**. Reaction conditions: ebullated bed, 15 mL of glycerol aqueous solution (0.1 mol·L^{-1}), glycerol/Pt (mol/mol) = 1000, 60 °C, O_2 30 mL/min, 8 h.

The reusability of 0.9%Pt_1+Pt_n/Cu-CuZrOx was tested in a glycerol/Pt (mol/mol) of 1000. After five runs, the glycerol conversion and the GLYA selectivity are well preserved at 79.8% and 84.4%, respectively (**Supplementary Fig. 16a**). Almost no leaching of Pt from solid catalyst or the spent reaction solution was detected (**Supplementary Fig. 16b**). While 4.7% and 3.8% of Cu and Zr dosages were detected to leach from the solid in five runs, which is close to the total loss of 4.2% and 3.6% in the reaction solution.

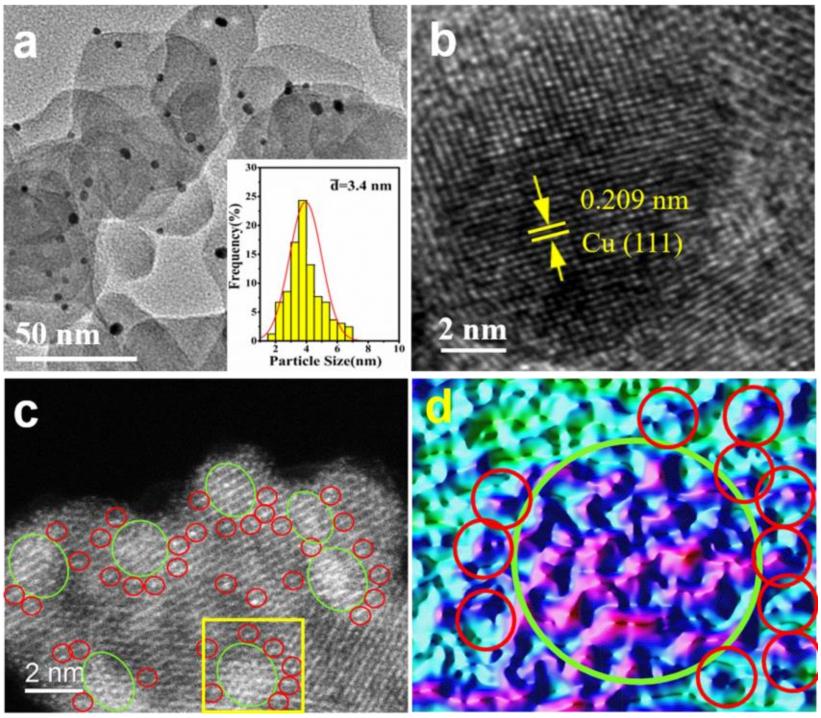

Supplementary Fig. 17 Dispersion structure of spent 0.9%Pt_1+Pt_n/Cu-CuZrO_x after five runs. **a,b** Representative HRTEM images and the corresponding particle size distribution. Scale bars: **a** 50 nm; **b** 2 nm. **c,d** Representative AC-HAADF-STEM images **(c)** and the corresponding 3D surface simulation of the marked regions in yellow rectangles **(d)** by the software Fiji.

In the HRTEM images of the spent 0.9%Pt_1+Pt_n/Cu-CuZrO_x (**Supplementary Fig. 17a and 17b**), the dispersion of PtCu nanoparticles is well retained, with Cu (111) plane in a maximum size distribution of around 3.4 nm similar to the fresh catalyst. The AC-HAADF-STEM images and the 3D surface simulation (**Supplementary Fig. 17c and 17d**) verify the coexistence of Pt_1 and Pt_n sites as well as the surrounding spatial distribution identical to the fresh catalyst.

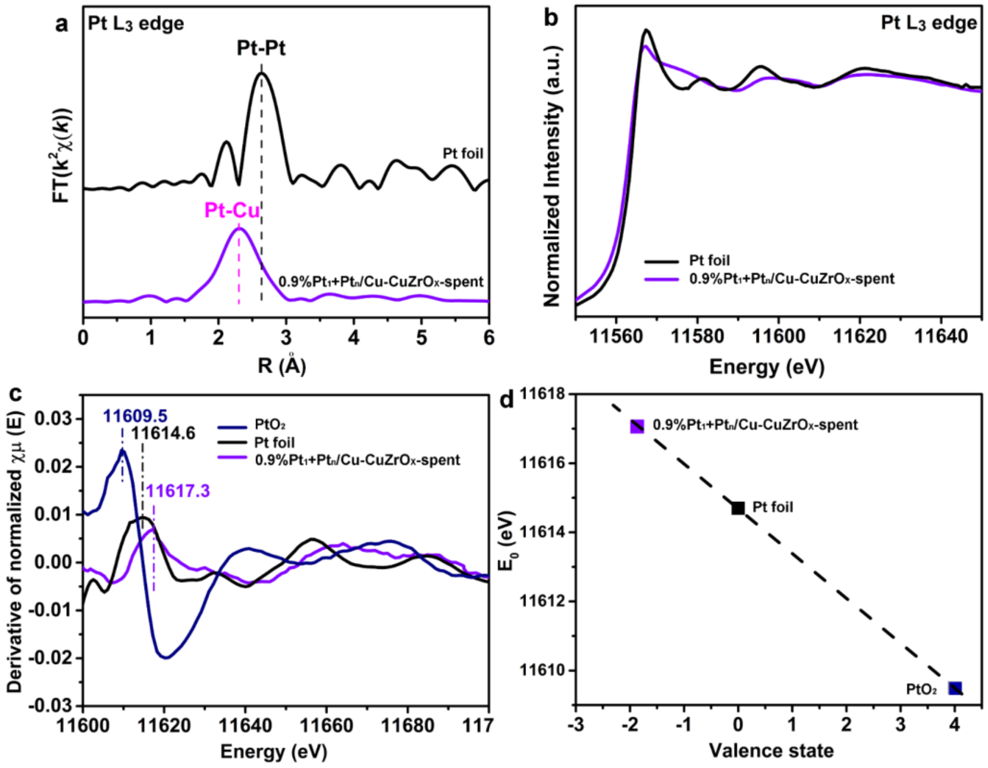

Supplementary Fig. 18 Coordination structure and electronic state of spent 0.9%Pt_1+Pt_n/Cu-CuZrO$_x$ after five runs. a,b Pt L3-edge FT-EXAFS spectra (**a**) and normalized XANES spectra (**b**). **c,d** First-derivative Pt L3-edge XANES spectra (**c**) and the valence state analysis (**d**).

Pt L3-edge FT-EXAFS spectra of the spent 0.9%Pt_1+Pt_n/Cu-CuZrO$_x$ (**Supplementary Fig. 18a**) displays identical Pt-Cu and Pt-Pt coordination with fitted 6.1 Pt-Cu coordination and 3.0 Pt-Pt coordination (**Supplementary Table 2, entry 7**). According to the normalized XANES spectra and the corresponding valence analysis for the spent 0.9%Pt_1+Pt_n/Cu-CuZrO$_x$ (**Supplementary Fig. 18b-d**), the $Pt^{\delta-}$ valence state has been quantified in a δ value of 1.85, same as the fresh one.

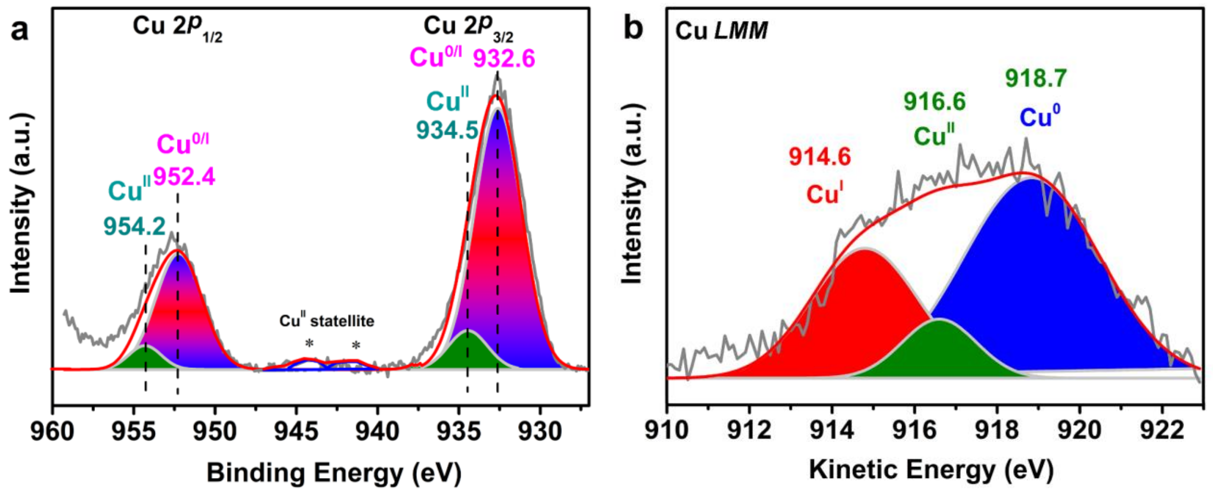

Supplementary Fig. 19 Electronic structure analysis of spent 0.9%Pt_1+Pt_n/Cu-CuZrO_x. Cu 2*p* core-level XPS spectra **(a)** and X-ray induced Cu Auger electron spectra **(b)** of spent 0.9%Pt_1+Pt_n/Cu-CuZrO_x after five runs. Reaction conditions: ebullated bed, 15 mL of glycerol aqueous solution (0.1 mol·L^{-1}), glycerol/Pt (mol/mol) = 1000, 60 ºC, O_2 30 mL/min, 8 h.

In the Cu XPS 2*p*3/2 spectra (**Supplementary Fig. 19a**), two binding energies assigned[1] to Cu^{II} and $Cu^{0/I}$ species are observed in the spent 0.9%Pt_1+Pt_n/Cu-CuZrO_x after five runs at 934.5 and 932.6 eV, which is as same as that of the fresh sample (**Supplementary Fig. 3a**). Similar results are observed in the X-ray induced Cu Auger electron spectra (**Supplementary Fig. 19b**). After the reaction, the surface Pt/Cu molar ratio increases from 1/15 in the fresh 0.9%Pt_1+Pt_n/Cu-CuZrO_x to 1/14 in the spent sample and the Cu^{II}/$Cu^{0/I}$ molar ratio decreases from 1/4 to 1/5. Considering no Pt leaching is detected (**Supplementary Fig. 16b**), Cu leaching are deduced to be originated from the Cu^{II} species.

II. Supplementary Tables

Supplementary Table 1. Physicochemical parameters of samples.

	Sample	BET (m^2/g)	Cu (wt%)[a]	Pt (wt%)[a]	Pt/Cu		Cu Mean Size (nm)[b]
					Bulk[a]	Surface[b]	
1	$Cu_1Zr_{4.3}O_{9.6}$	60	10.4	-	-	-	-
2	Cu-$CuZrO_x$	57	10.6	-	-	-	3.4
3	0.5%Pt_1/Cu-$CuZrO_x$	56	9.5	0.5	1/54	1/30	3.3
4	0.9%Pt_1+Pt_n/Cu-$CuZrO_x$	57	8.2	0.9	1/34	1/15	3.4
5	0.9%Pt_n/Cu-$CuZrO_x$	57	10.0	0.9	1/34	1/17	3.4
6	0.9%PtCu-$CuZrO_x$	60	10.1	0.9	1/34	1/14	3.5
7	0.9%Pt_1+Pt_n/Cu-$CuZrO_x$-spent[c]	60	7.9	0.9	1/33	1/14	3.4

[a] Determined by ICP-AES. [b] Determined by HRTEM. [c] Used in five runs.

Supplementary Table 2. Structural parameters of supported PtCu catalysts extracted from the EXAFS fitting with references of bulk Pt foil and PtO_2.

	Samples	Shell	N[a]	R (Å)[b]	σ^2 ($Å^2 \cdot 10^{-3}$)[c]	ΔE_0 (eV)[d]	R factor (%)[e]
1	Pt foil	Pt-Pt	12	2.77	5.6	2.9	0.11
2	PtO_2	Pt-O	6.0	2.05	4.4	-3.6	0.24
		Pt-Pt	6.0	3.09	5.1	-6.6	
3	0.5%Pt_1/Cu-CuZrO_x	Pt-Cu	8.3	2.61	6.7	7.2	0.50
4	0.9%Pt_1+Pt_n/Cu-CuZrO_x	Pt-Cu	6.1	2.62	10.0	7.6	0.40
		Pt-Pt	3.0	2.65	2.9	7.4	
5	0.9%Pt_n/Cu-CuZrO_x	Pt-Cu	4.1	2.60	7.2	7.5	0.41
		Pt-Pt	2.9	2.76	8.7	10.1	
6	0.9%PtCu-CuZrO_x	Pt-Cu	4.1	2.60	7.2	7.5	0.40
		Pt-Pt	4.3	2.72	2.8	7.4	
7	0.9%Pt_1+Pt_n/Cu-CuZrO_x-spent[f]	Pt-Cu	6.1	2.77	6.3	-7.6	0.40
		Pt-Pt	3.0	2.58	4.2	8.4	

[a] N: coordination number; [b] R: bond distance; [c] σ^2: Debye-Waller factors; [d] ΔE_0: the inner potential correction; [e] R factor: if r <5%, consistent with broadly correct models. S_0^2 was set as 0.9 for Pt-Pt/Pt-Cu, which were obtained from the experimental EXAFS fit of Pt foil reference by fixing N as the known crystallographic value and was fixed to all the samples. [f] Used in five runs.

Supplementary Table 3. Band assignments upon the adsorption of 1-propanol on supported PtCu catalysts. [a]

	Adsorption species	Adsorption model	Characteristic frequency (cm^{-1})			
1	Adsorbed bidentate 1-propanol	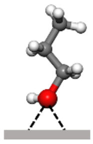	1066<br>1229	ν(C-OH)<br>δ(COH)		
2	Adsorbed bidentate propoxy	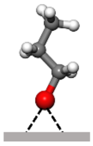	1053	ν(C-O)	2971<br>2941<br>2882<br>1473<br>1458<br>1401<br>1387<br>1099<br>1013	$\nu_a(CH_3)$<br>$\nu_a(CH_2)$<br>$\nu_s(CH_2)$<br>$\delta_a(CH_3)$<br>$\delta(CH_2)$-*s*<br>$\delta_s(CH_3)$<br>$\omega(CH_2)$<br>coupling of $\rho(CH_3)$ and ν(CC)<br>ν(CCC)
3	Adsorbed monodentate propoxy	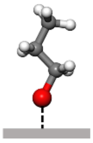	1138	ν(C-O)		
4	η^1-adsorbed propanal	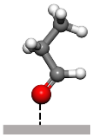	1670<br>1340	ν(C=O)<br>δ(CH)		
5	η^2-adsorbed propanal	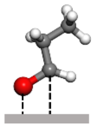	2740<br>1275<br>1350	ν(CH)<br>ν(C-O)<br>δ(CH)		

[a] Band assignments are identified according to the references[4-6].

Supplementary Table 4. Band assignments upon the adsorption of glycerol on supported PtCu catalysts.[a]

	Adsorption species	Adsorption model	Characteristic frequency (cm^{-1})			
1	Non-dissociated glycerol	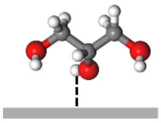	1040<br>1152	Primary v(C-OH)<br>H-bonded Secondary v(C-OH)	2926<br>2853<br>1474<br>1410<br>1300<br>1260	$v_a(CH_2)$<br>$v_s(CH_2)$<br>$\delta(CH_2)$<br>δ(OH)<br>$\tau(CH_2)$<br>$\omega(CH_2)$
2	Monodentate dissociated glycerol	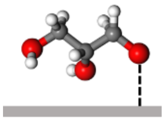	1054<br>1099	Primary v(C-O)<br>Secondary v(C-OH)		
3	Bidentate dissociated glycerol	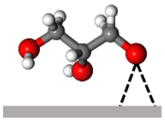	1068<br>1099	Primary v(C-O)<br>Secondary v(C-OH)		
4	η[1]-adsorbed GLAD	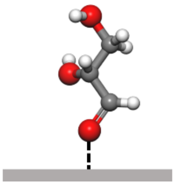	1628	v(C=O)	2966<br>1430<br>1387	$v_a(CH_2)$<br>δ(OH)<br>ρ(OH)
5	η[2]-adsorbed GLAD	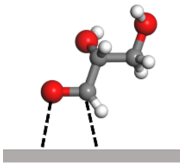	2713<br>1337<br>1186	v(CH)<br>δ(CH)<br>v(C-O)		
6	Adsorbed glycerate	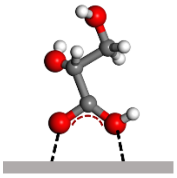	1590, 1509	v(OCO)		

[a] Band assignments are identified according to the FT-IR spectra of pristine glycerol (Supplementary Fig.10a) and the references[7-12].

Supplementary Table 5. Band assignments upon the adsorption of acetaldehyde on supported PtCu catalysts.[a]

	Adsorption species	Adsorption model	Characteristic frequency (cm^{-1})	
1	Adsorbed bidentate ethoxy	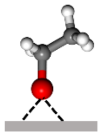	1101 *v*(C-O)	2965 $v_a(CH_3)$<br>2940 $v_a(CH_2)$
2	Adsorbed monodentate ethoxy	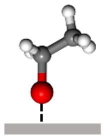	1125 *v*(C-O)	2884 $v_s(CH_3)$<br>1408 $\delta(CH_3)$
3	η^1-adsorbed acetaldehyde	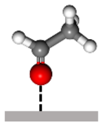	2703 *v*(CH)<br>1760-1730 *v*(C=O)<br>1340 δ(CH)	
4	η^2-adsorbed acetaldehyde	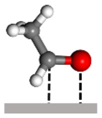	2735 *v*(CH)<br>1275 *v*(C-O)<br>1352 δ(CH)<br>1345 δ(CH)<br>1178 *v*(CC)	
5	Adsorbed acetate	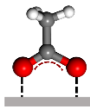	1548, 1459 *v*(OCO)	

[a] Band assignments are identified according to the references[13-15].

Supplementary Table 6. Band assignments upon the adsorption of glyceraldehyde on supported PtCu catalysts.[a]

<table>
<tr><th></th><th>Adsorption species</th><th>Adsorption model</th><th colspan="2">Characteristic frequency (cm^{-1})</th></tr>
<tr><td>1</td><td>η^1-adsorbed GLAD</td><td>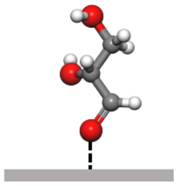</td><td>1646 ν(C=O)
1624 ν(C=O)
1395 ρ(OH)</td><td rowspan="2">2962 $\nu_a(CH_2)$
2882 $\nu_s(CH_2)$
1463 $\delta(CH_2)$
1426 δ(OH)
1303 $\tau(CH_2)$
1230 $\omega(CH_2)$
1146 Secondary ν(C-OH)
1075 Primary ν(C-OH)</td></tr>
<tr><td>2</td><td>η^2-adsorbed GLAD</td><td>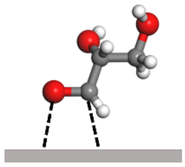</td><td>2713 ν(CH)
1370 ρ(OH)
1196/1183[b] ν(C-O)</td></tr>
<tr><td>3</td><td>Adsorbed glycerate</td><td>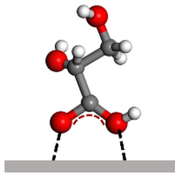</td><td>1588, 1530 ν(OCO)</td><td></td></tr>
</table>

[a] Band assignments are identified according to the FT-IR spectra of pristine glyceraldehyde (Supplementary Fig.10b) and the references[7-12]. [b] 1196 cm^{-1} on 0.9%Pt_1+Pt_n/Cu-$CuZrO_x$ and 1183 cm^{-1} on 0.9%Pt_n/Cu-$CuZrO_x$.

Supplementary Table 7. Band assignments upon the adsorption of propionic acid on supported PtCu catalyst. [a]

	Adsorption species	Adsorption model	Characteristic frequency (cm^{-1})	
1	Adsorbed monodentate propionic acid	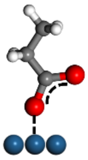	1597 ν(OCO)<br>1728, 1715 ν(C=O)	2990 $\nu_a(CH_3)$<br>2946 $\nu_a(CH_2)$<br>2915 $\nu_s(CH_3)$<br>2884 $\nu_s(CH_2)$<br>1470 $\delta_a(CH_3)$
2	Adsorbed bidentate propionic acid	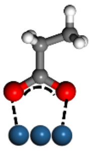	1540-1530 ν_a(OCO)<br>1440-1430 ν_s(OCO)<br>1728, 1715 ν(C=O)	1415 $\delta_s(CH_3)$<br>1378 δ(CH)<br>1243 δ(OH)<br>1019 $\rho(CH_3)$
3	Propionic acid vapor	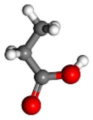	1790, 1775, 1730 ν(C=O)<br>1145 ν(C-O)	2992 $\nu_a(CH_3)$<br>2954 $\nu_a(CH_2)$<br>2903 $\nu_s(CH_2)$<br>1473, 1421 $\delta_a(CH_3)$<br>1384, 1335 $\delta_s(CH_3)$<br>1292 δ(CH)<br>1247 δ(OH)<br>1073 $\rho(CH_3)$

[a] Band assignments are identified according to the references[6,15-17].

Supplementary Table 8. Catalytic performance toward the selective oxidation of glycerol over varied catalysts.[a]

	Catalyst	Glycerol conversion (%)	GLYA yield (%)[b]	Product selectivity (%)[c]								C-balance (%)[d]
				GLYA	GLAD	DHA	TTA	OA	FA	GCA	HAc	
1	Cu-CuZrO_x	0.4	0	0	0	0	0	47.5	35.3	0	0	82.8
2	0.5%Pt_1/Cu-CuZrO_x	3.7	2.4	64.1	14.2	7.7	0.6	1.2	0	0.8	8.8	97.4
3	0.9%Pt_1+Pt_n/Cu-CuZrO_x	90.1<br>(89.9)	72.0<br>(72.3)	80.0<br>(80.4)	4.6<br>(5.2)	10.5<br>(9.5)	1.4<br>(2.4)	1.2<br>(1.3)	0<br>(0)	1.1<br>(1.0)	0.7<br>(1.1)	99.5<br>(100.9)
4	0.9%Pt_n/Cu-CuZrO_x	85.6	57.3	66.9	10.6	11.9	1.9	0.9	0	1.2	5.4	98.8
5	0.9%PtCu-CuZrO_x	65.0	39.4	60.6	9.5	12.6	2.0	1.2	0	1.3	9.6	96.8
6	0.9%Pt_1+Pt_n/Cu-CuZrO_x-N_2O	75.0	50.9	67.9	18.2	9.9	1.4	0.9	0	0.8	0.1	99.2

[a] Reaction conditions: ebullated bed, 15 mL of glycerol aqueous solution (0.1 mol·L^{-1}), glycerol/Pt (mol/mol) = 300, 60 °C, O_2 30 mL/min, 8 h. [b] GLYA yield = (measured moles of GLYA/theoretical moles of GLYA) × 100 %. [c] Abbreviations: GLYA (glyceric acid), GLAD (glyceraldehyde), DHA (dihydroxyacetone), TTA (tartronic), OA (oxalic acid), FA (formic acid), GCA (glycolic acid), and HAc (acetic acid). [d] C-balance was calculated based on the liquid products. CO_2 was identified as the only gaseous product.

Supplementary Table 9. Parameters used in the Weisz-Prater criterion and Mears Criterion for estimating mass transfer limitations in glycerol oxidation.

**Parameters**	**0.9%Pt₁+Ptₙ/Cu-CuZrOₓ**	**0.9%Ptₙ/Cu-CuZrOₓ**	**0.9%PtCu-CuZrOₓ**
Reaction rate: $-r'_A$ ($kmol/kg_{cat}$ s)[a]	2.05×10^{-6}	1.54×10^{-6}	7.70×10^{-7}
Density of catalyst: ρ_b (kg/m^3)	1775	1780	1788
Primary particle size: R_1 (m)[b]	$<1.0\times10^{-7}$	$<1.0\times10^{-7}$	$<1.0\times10^{-7}$
Secondary particle size: R_2 (m)[c]	$<6.617\times10^{-6}$	$<6.314\times10^{-6}$	$<9.733\times10^{-6}$
Concentration of glycerol at 60 °C: C_{Ab} ($kmol/m^3$)[d]	0.1	0.1	0.1
Effective liquid-phase diffusivity: D_e (m^2/s)[e]	7.0×10^{-11}	6.7×10^{-11}	6.6×10^{-11}
Porosity: ϕ_p[f]	0.110	0.106	0.103
Tortuosity: $1-0.5ln\phi_p$	2.104	2.121	2.134
Reaction order: n	~1	~1	~1
Mass transfer coefficient: k_c (m/s)	3.11×10^{-3}	3.11×10^{-3}	3.11×10^{-3}
Sherwood number: Sh[g]	2.011	2.011	2.011
Reynolds number: Re[h]	2.59×10^{-6}	2.59×10^{-6}	2.59×10^{-6}
Schmidt number: Sc[i]	1.6×10^{6}	1.6×10^{6}	1.6×10^{6}
Liquid-phase diffusivity: D_{AB} (m^2/s)[j]	$>0.309\times10^{-9}$	$>0.309\times10^{-9}$	$>0.309\times10^{-9}$
Superficial velocity: U (m/s)	6.4×10^{-3}	6.4×10^{-3}	6.4×10^{-3}
Density of reactant mixture fluid at 60 °C: ρ (kg/m^3)[d]	~1.01	~1.01	~1.01
Viscosity of the reactant mixture fluid at 60 °C: μ (Pa·s)[k]	$<0.50\times10^{-3}$	$<0.50\times10^{-3}$	$<0.50\times10^{-3}$
**Mears Criterion for External Diffusion:**			
$\frac{-r'_A\rho_b R_2 n}{k_c C_{Ab}}$	**7.74×10^{-5}**	**5.57×10^{-5}**	**4.31×10^{-5}**
**Weisz-Prater Criterion for Internal Diffusion:**			
$C_{wp}=\frac{-r'_A\rho_b R_1^2}{C_{Ab}D_e}$	**2.96×10^{-9}**	**2.32×10^{-9}**	**1.18×10^{-9}**

[a] Calculated at < 20% glycerol conversion. Glycerol/Pt (mol/mol) = 300, 60 °C, O_2 30 mL/min. [b] Calculated from TEM characterization. [c] Determined by the laser particle size analyzer. [d] Calculated based on liquid component. [e] $D_e=\frac{D_{AB}\phi_p\sigma_c}{\tau}$, where ϕ_p is porosity, σ_c is constriction factor, and τ is tortuosity. σ_c is taken as 1 for many cases according to ref. 18. f Determined by the N_2 adsorption-desorption experiments. [g] $Sh=\frac{2k_cR}{D_{AB}}=2+0.6Re^{\frac{1}{2}}Sc^{\frac{1}{3}}$. [h] $Re=\frac{2UR\rho}{\mu}$. [i] $Sc=\frac{\mu}{\rho D_{AB}}$. [j] Calculated based on the ref. 19. [k] Calculated based on Wilke formula.

According to the references[20,21], since $\frac{-r_A'\rho_b Rn}{k_c C_{Ab}}<0.15$, the external mass transfer effects could be neglected. Since $C_{wp}=\frac{-r'_A\rho_b R_1^2}{C_{Ab}D_e}<1$, the internal mass transfer effects could be neglected.

Supplementary Table 10. Catalytic performance toward the selective oxidation of glycerol over 0.9%Pt_1+Pt_n/Cu-CuZrO_x under tailored reaction condition.[a]

	Glycerol Concentration (mol/L)	Glycerol/Pt (mol/mol)	O_2 flow rate (mL/min)	Glycerol conversion (%)	Reaction rate (mol_{gl} • mol_{Pt}^{-1} • h^{-1})[b]	GLYA yield (%)[c]	Product selectivity (%)[d]								C-balance (%)[e]
							GLYA	GLAD	DHA	TTA	OA	FA	GCA	HAc	
1	0.1	300	30	90.1	160	72.0	80.0	4.6	10.5	1.4	1.2	0	1.1	0.7	99.5
				(89.9)	158	(72.3)	(80.4)	(5.2)	(9.5)	(2.4)	(1.3)	(0)	(1.0)	(1.1)	(100.9)
2	0.1	300	60	90.0	162	72.1	80.1	4.7	10.2	1.8	1.2	0	1.0	0.8	99.8
3	0.1	300	150	91.0	161	72.5	79.3	5.0	9.8	2.1	2.0	0	0.8	0.5	99.5
4	0.1	500	30	85.1	158	68.9	81.0	4.2	9.5	1.4	1.0	0	1.3	0.5	98.9
5	0.1	650	30	82.2	160	67.8	82.5	4.0	8.6	1.5	1.1	0	1.0	0.9	99.6
6	0.1	1000	30	79.8	157	67.4	84.4	3.3	7.5	1.0	1.0	0	0.6	0.7	98.5
7	0.3	300	30	80.0	158	65.8	82.3	4.5	10.0	2.0	1.4	0	0	0.3	100.5

[a] Reaction conditions: 0.9%Pt_1+Pt_n/Cu-CuZrO_x as catalyst, ebullated bed, 15 mL of glycerol aqueous solution, 60 °C, O_2 flow, 8 h. [b] calculated at the initial reaction with a glycerol conversion of < 20%. [c] GLYA yield = (measured moles of GLYA/theoretical moles of GLYA) × 100 %. [d] Abbreviations: GLYA (glyceric acid), GLAD (glyceraldehyde), DHA (dihydroxyacetone), TTA (tartronic), OA (oxalic acid), FA (formic acid), GCA (glycolic acid), and HAc (acetic acid). [e] C-balance was calculated based on the liquid products. CO_2 was identified as the only gaseous product.

Supplementary Table 11. Comparison studies on catalytic performance toward glycerol oxidation to GLYA.

	**Catalyst**	**T** (°C)	**O_2**		**Pt loading** (wt%)	**Glycerol Concentration (mol/L)**	**Glycerol/Pt (mol/mol)**	**Time** (h)	**Glycerol conversion** (%)	**GLYA selectivity** (%)	**GLYA yield** (%)	**Ref.**
			P^{a} (MPa)	R_{Stream}^{b} (mL/min)								
1	0.9%Pt_1+Pt_n/Cu-$CuZrO_x$	60	-	30	0.9	0.1	300	8	90.1 (89.9)	80.0 (80.4)	72.0 (72.3)	This work
						0.3	300	15	80.0	82.3	65.8	
						0.1	1000	15	79.8	84.4	67.4	
2	PtCu/CNT	60	-	150	5.0	1.1	200	6	86.2	70.8	61.0	[22]
3	PtSn/AC	60	-	15	2.0	0.5	1000	8	91.1	55.4	50.5	[23]
4	AuPt/MgO	23	0.3	-	1.0	0.3	500	24	42.5	85.1	36.2	[24]
5	PtCo/RGO	60	-	30	10	1.1	440	3	70.2	85.9	60.3	[25]
6	Pt-CeO_2/CNT	60	-	150	5.0	0.5	200	6	86.0	71.0	61.1	[26]
7	AuPt/CeO_2	80	0.1	-	2.0	0.3	267	2	85.1	60.5	51.5	[27]
8	PtCo/MCM-41	60	1	-	0.6	0.2	530	8	55.4	79.0	43.8	[28]
9	$Pt_{60}Au_{40}$/HT	25	-	10		0.25	287	6	73.0	78.0	56.9	[29]
10	Pt/NG-MWCNTs	60	0.5	-	3.6	1.1	600	3	64.4	81.0	52.2	[30]
11	PtZn-$ZnTiO_x$/$ZnTiO_3$	80	0.1	-	1.5	0.3	500	8	80.6	65.4	52.7	[12]
12	AuPt(6:4)/H-mordenite	100	0.3	-	1.0	0.3	500	2	70.0	83.0	58.1	[31]

[a] O_2 pressure in sealed batch reactor, [b] O_2 stream rate in flowing reactor.

III. Supplementary Methods

1. The details of galvanic replacement method

A galvanic replacement method was employed to introduce Pt atoms onto the Cu surface. As the standard reduction potential of $PtCl_6^{2-}$/Pt redox pair (1.44 V vs. the standard hydrogen electrode (SHE)) is much higher than that of Cu^{2+}/Cu redox pair (0.34 V vs. SHE), the following thermodynamically-favourable redox reaction occurs:

$$2Cu^0\ (s) + PtCl_6^{2-}\ (aq) = Pt^0\ (s) + 2Cu^{2+}\ (aq) + 6Cl^-\ (aq) \qquad (1)$$

The large potential difference (up to 0.76 V) guarantees the reaction feasibility and makes this replacement reaction occur rapidly at room temperature. A thorough washing by deoxygenated deionized water has been conducted to completely remove the surface Cu^{2+} and Cl^- ions. Then a reduction step has been carried out in a H_2 stream at 450 ºC (heating rate: 5 ºC·min^{-1}) for 2 h, producing supported PtCu.

2. Analysis method for the reaction liquid

An Agilent LC-1260 high-performance liquid chromatograph (HPLC) equipped with both an ultraviolet (UV, 210 nm) detector and a refractive index detector (RID) has been used for the analysis of the reaction liquid after catalysts separation by filtration. An Aminex HPX-87 H column (Bio-Rad, 300×7.8 mm) operating at 50 ºC was used with 10 mM aqueous H_2SO_4 or HCOOH as eluents at a flow of 0.5 mL·min^{-1}. The retention time of each compound in HPLC was determined by comparing with the corresponding pure sample.

Glyceric acid (GLYA) and glyceraldehyde (GLAD) were separated and quantified based on HPLC data using HCOOH eluent in UV detector. Dihydroxyacetone (DHA), glycolic acid (GCA), oxalic acid (OA), tartronic acid (TTA), acetic acid (HAc), and formic acid (FA) were separated and quantified based on HPLC data using H_2SO_4 eluent in UV detector. Because glycerol could not be detected by UV detector, a RID detector using H_2SO_4 eluent was employed, giving a total amount of glycerol and DHA. Then the amount of glycerol was calculated by subtracting the amount of DHA determined in UV detector.

Glycerol conversion, carbon balance, product selectivity, and GLYA yield were calculated by the following equations:

$$Glycerol\ conversion = \left(1 - \frac{moles\ of\ glycerol}{moles\ of\ glycerol\ loaded\ initially}\right) \times 100\ \%\ ,$$

$$Carbon\ balance = \frac{\sum_{i=1}^{3}\ i \times moles\ of\ Ci\ product}{3 \times moles\ of\ glycerol\ converted} \times 100\ \%\ ,$$

$$Product\ Selectivity = \frac{i \times moles\ of\ Ci\ product}{\sum_{i=1}^{3}\ i \times moles\ of\ Ci\ product} \times 100\ \%\ ,$$

$$GLYA\ yield = \frac{measured\ moles\ of\ GLYA}{theoretical\ moles\ of\ GLYA} \times 100\ \%$$

(1) HPLC data using HCOOH in UV detector and the corresponding calibration curves

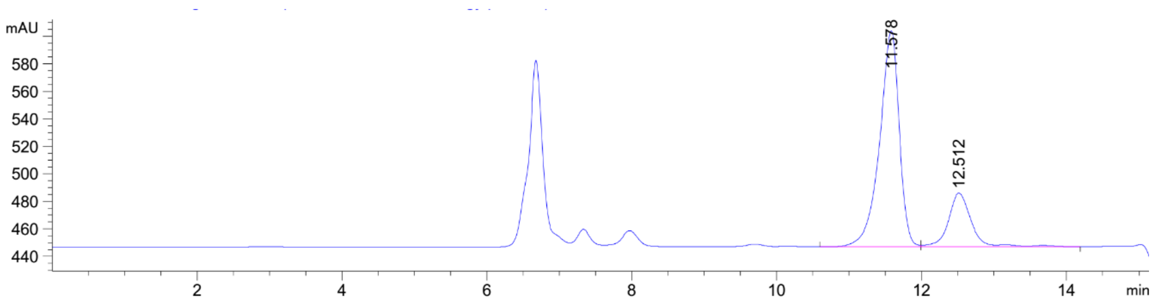

Retention time (min)	Product
6.981	H_2O
11.578	GLYA
12.512	GLAD

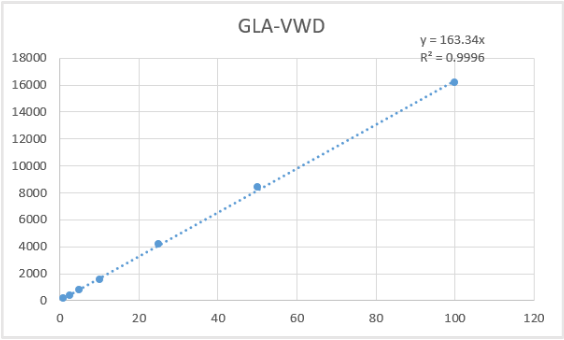

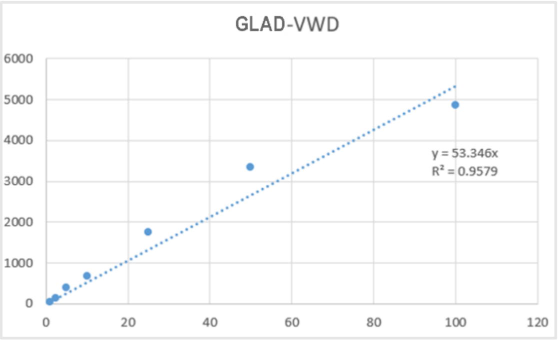

(2) HPLC data using H_2SO_4 in UV detector and the corresponding calibration curves

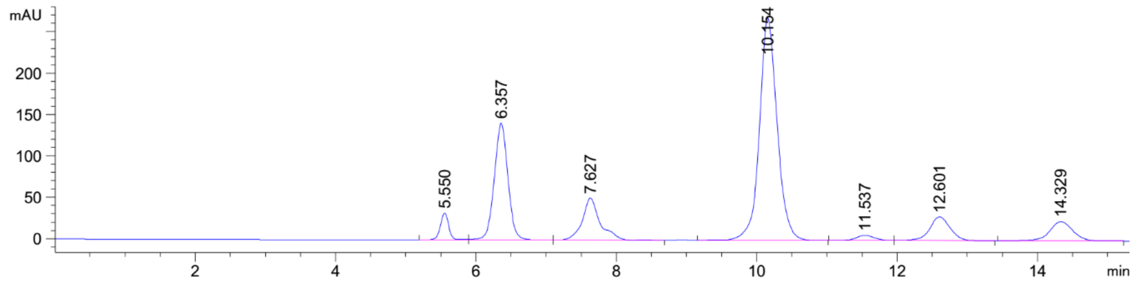

Retention time (min)	Product
5.550	H_2O
6.357	OA
7.627	TTA
10.154	GLYA /GLAD
11.537	GCA
12.601	DHA
14.329	HAc

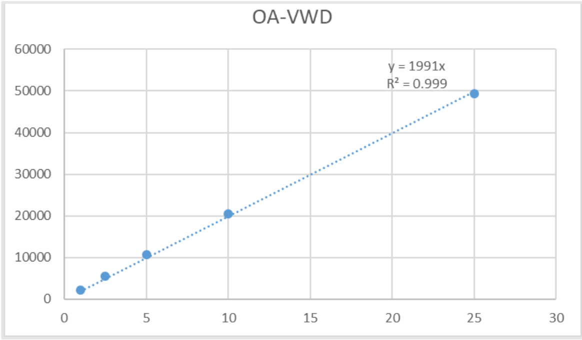

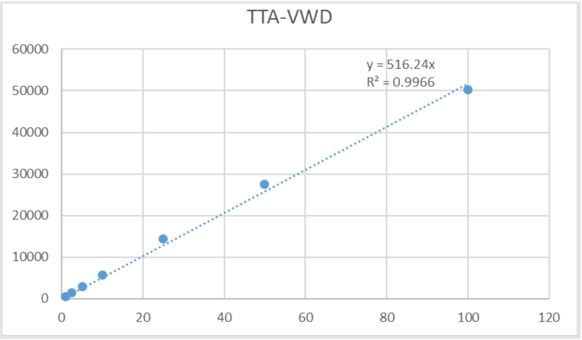

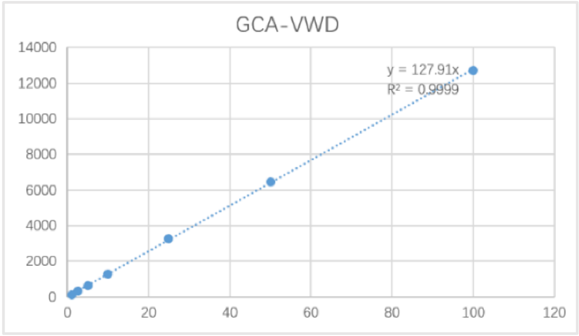

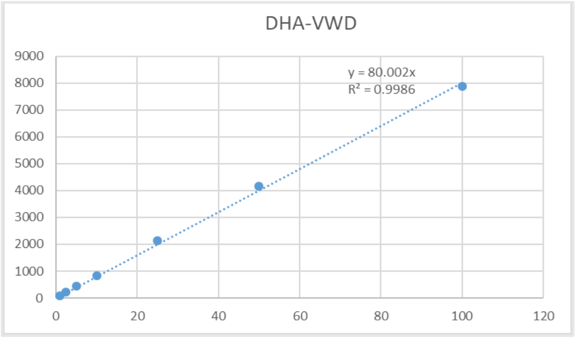

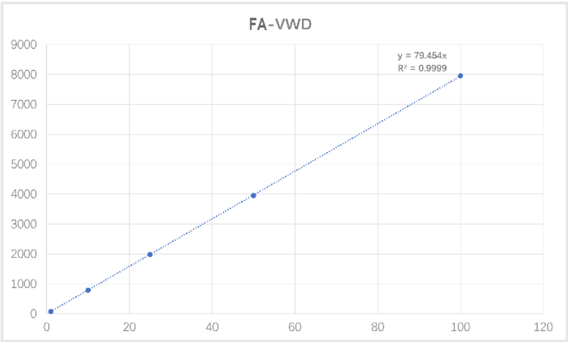

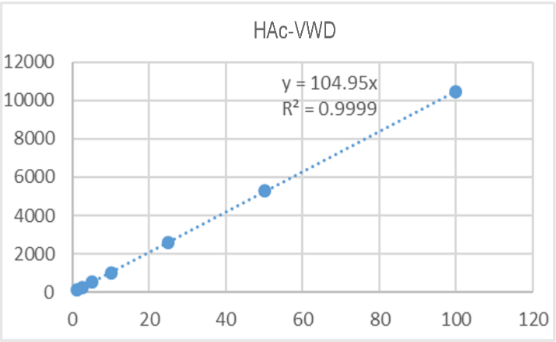

(3) HPLC data using H_2SO_4 in RID and the corresponding calibration curves

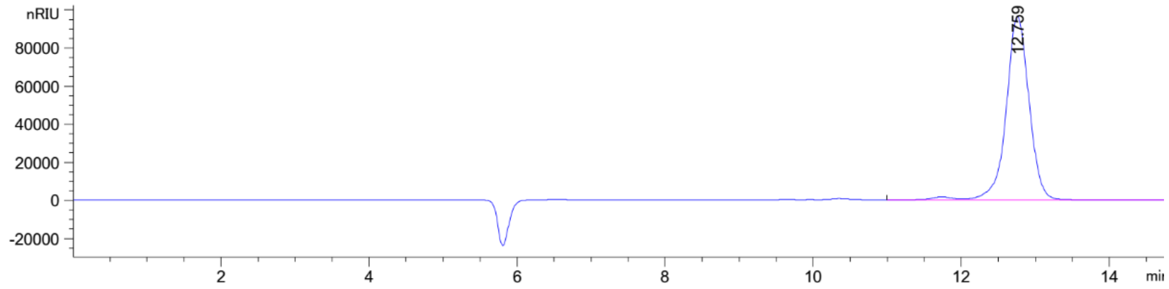

Retention time (min)	Product
12.769	Glycerol + DHA

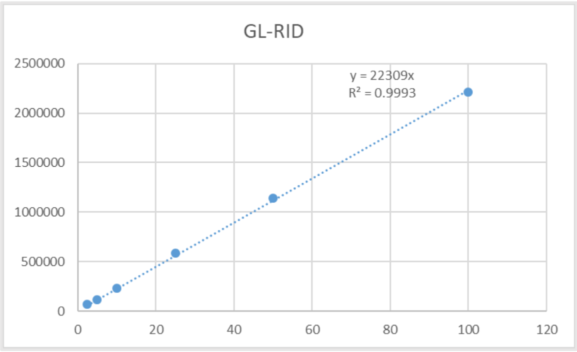

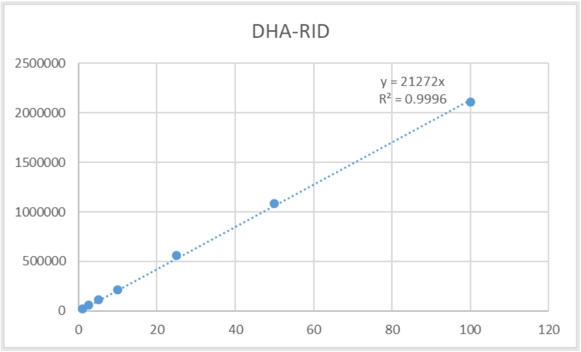

3. Analysis method for the gaseous product

A Shimadzu 2014C GC equipped with a TDX-1 column and TCD detector has been used for the identification of the collected gaseous product. CO_2 was identified as the only gas product of the reactions.

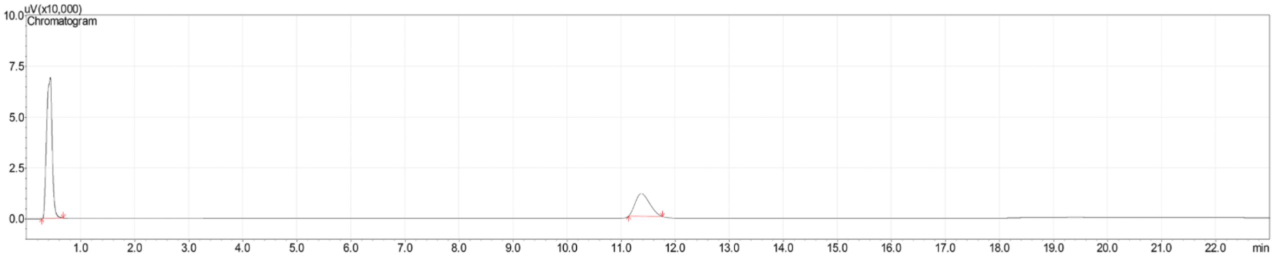

Retention time (min)	Product	f_i
1.5	N_2	1.00
11.4	CO_2	1.27

V. Supplementary References

1. Zheng, X., Lin, H., Zheng, J., Duan, X. & Yuan, Y. Lanthanum oxide-modified Cu/SiO_2 as a high-performance catalyst for chemoselective hydrogenation of dimethyl oxalate to ethylene glycol. *ACS Catal.* **3**, 2738-2749 (2013).
2. Wang, Q. et al. Insight into the effect of dual active Cu^0/Cu^+ sites in a $Cu/ZnO-Al_2O_3$ catalyst on 5-hydroxylmethylfurfural hydrodeoxygenation. *ACS Sustain. Chem. Eng.* **8**, 15288-15298 (2020).
3. Du, L. M., Zeng, H., Lu, S. M. & Fang, B. S. Studies on structures and properties of glycerol and 3-hydroxypropionaldehyde by density function theory method. *J. Fuzhou Univ. Nat. Sci. Ed.* **39**, 594-598 (2011).
4. Street, S. C. & Gellman, A. J. FT-IRAS of adsorbed alkoxides: 1-propoxide on Cu(111). *Surf. Sci.* **372**, 223-238 (1997).
5. Myint, M., Yan, Y. & Chen, J. G. Reaction pathways of propanal and 1-propanol on Fe/Ni(111) and Cu/Ni(111) Bimetallic Surfaces. *J. Phys. Chem. C* **118**, 11340-11349 (2014).
6. Kammert, J. D., Chemburkar, A., Miyake, N., Neurock, M. & Davis, R. J. Reaction kinetics and mechanism for the catalytic reduction of propionic acid over supported ReO_x promoted by Pd. *ACS Catal.* **11**, 1435-1455 (2021).
7. Foo, G. S., Wei, D., Sholl, D. S. & Sievers C. Role of lewis and brønsted acid sites in the dehydration of glycerol over Niobia, *ACS Catal.* **4**, 3180-3192 (2014).
8. Zhang, X. et al. Platinum–copper single atom alloy catalysts with high performance towards glycerol hydrogenolysis. *Nat. Commun.* **10**, 5812 (2019).
9. Gomes J. F. et al. Influence of silver on the glycerol electro-oxidation over AuAg/C catalysts in alkaline medium: a cyclic voltammetry and *in situ* FTIR spectroscopy study. *Electrochimica Acta* **144,** 361-368 (2014).
10. Copeland, J. R. et al. Surface interactions of glycerol with acidic and basic metal oxides. *J. Phys. Chem. C*, **117**, 21413-21425 (2013).
11. Yfanti, V. -L. & Lemonidou, A. A. Mechanistic study of liquid phase glycerol hydrodeoxygenation with *in-situ* generated hydrogen. *J. Catal.* **368**, 98-111 (2018).
12. Zhang, Y., Zhang, X., Yang, P., Gao, M., Feng, J., Li, D. *In situ* topologically induced PtZn alloy-$ZnTiO_x$ and the synergistic effect on glycerol oxidation. *Appl. Catal. B: Environ.* **298**, 120634 (2021).
13. Ochoa, J. V., Trevisanut, C., Millet, J.-M. M., Busca, G. & Cavani, F. *In situ* DRIFTS-MS study of the anaerobic oxidation of ethanol over spinel mixed oxides. *J. Phys. Chem. C* **117**, 23908-23918 (2013).
14. Mann, A. K. P., Wu, Z., Calaza, F. C. & Overbury, S. H. Adsorption and reaction of acetaldehyde on shape-controlled CeO_2 nanocrystals: elucidation of structure-function relationships. *ACS Catal.* **4**, 2437-2448 (2014).
15. Yee, A., Morrison, S. J. & Idriss, H. A study of the reactions of ethanol on CeO_2 and Pd/CeO_2 by steady state reactions, ttemperature programmed desorption, and *in situ* FT-IR. *J. Catal.* **186**, 279-295 (1999).
16. Pei, Z.-F. & Ponec, V. On the intermediates of the acetic acid reactions on oxides: an IR study. Appl. Surf. Sci. 103, 171-182 (1996).
17. Ding, S., Wang, H., Han, J., Zhu, X. & Ge, Q. Ketonization of Propionic Acid to 3-Pentanone over

$Ce_xZr_{1-x}O_2$ Catalysts: The Importance of Acid–Base Balance. *Ind. Eng. Chem. Res.* **57**, 17086-17096 (2018).

18. Thirumal bai, P., Manokarana, V., Saiprasad, P. S. & Srinath, S. Studies on heat and mass transfer limitations in oxidative dehydrogenation of ethane over Cr_2O_3 /Al_2O_3 catalyst. *Procedia Engineering* **127**, 1338-1345 (2015).
19. D'Errico, G., Ortona, O., Capuano, F. & Vitagliano V. *J. Chem. Eng. Data* **49**, 1665-1670 (2004).
20. Pan, Y., Wu, G., He, Y., Feng, J. & Li D. Identification of the Au/ZnO interface as the specific active site for the selective oxidation of the secondary alcohol group in glycerol. *J. Catal.* **369**, 222-232 (2019).
21. Zope, B. N. & Davis, R. J. Influence of reactor configuration on the selective oxidation of glycerol over Au/TiO_2, *Top Catal.* **52**, 269-277 (2009).
22. Liang, D. et al. Bimetallic Pt-Cu catalysts for glycerol oxidation with oxygen in a base-free aqueous solution. *Catal. Commun.* **12**, 1059-1062 (2011).
23. Dou, J. et al. Carbon supported Pt9Sn1 nanoparticles as an efficient nanocatalyst for glycerol oxidation. *Appl. Catal. B: Environ.* **180**, 78-85 (2016).
24. Brett, G. L. et al. Selective oxidation of glycerol by highly active bimetallic catalysts at ambient temperature under base-free conditions, *Angew. Chem. Int. Ed.* **50**, 10136-10139 (2011).
25. Zhang, M., Shi, J., Ning, W. & Hou, Z. Reduced graphene oxide decorated with PtCo bimetallic nanoparticles: Facile fabrication and application for base-free oxidation of glycerol, *Catal. Today* **298**, 234-240 (2017).
26. Zhang, X. et al. Overcoming the deactivation of Pt/CNT by introducing CeO_2 for selective base-free glycerol-to-glyceric acid oxidation. *ACS Catal.* **10**, 3832-3837 (2020).
27. Zhang, X. et al. Support morphology effect on the selective oxidation of glycerol over AuPt/CeO_2 catalysts. *J. Catal.* **385**, 146-159 (2020).
28. Yan, H. et al. Enhanced performance of bimetallic PtCo/MCM-41 catalysts for glycerol oxidation in base-free medium. *Catal. Sci. Technol.* **9**, 4909-4919 (2019).
29. Tongsakul, D., Nishimura, S., Ebitani, K. Platinum/gold alloy nanoparticles-supported hydrotalcite catalyst for selective aerobic oxidation of polyols in base-free aqueous solution at room temperature. *ACS Catal.* **3**, 2199-2207 (2013).
30. Zhang, M., Sun, Y., Shi, J., Ning, W., Hou, Z. Selective glycerol oxidation using platinum nanoparticles supported on multi-walled carbon nanotubes and nitrogen-doped graphene hybrid. *Chinese J. Catal.* **38**, 537-544 (2017).
31. Villa, A., Veith, G. M. & Prati L. Selective oxidation of glycerol under acidic conditions using gold catalysts. *Angew. Chem. Int. Ed.* **49**, 4499-4502 (2010).
